# Alternative splicing in shaping the molecular landscape of the cochlea

**DOI:** 10.3389/fcell.2023.1143428

**Published:** 2023-03-02

**Authors:** Kwan Soo Kim, Hei Yeun Koo, Jinwoong Bok

**Affiliations:** ^1^ Department of Anatomy, Yonsei University College of Medicine, Seoul, Republic of Korea; ^2^ Brain Korea 21 Project for Medical Science, Yonsei University College of Medicine, Seoul, Republic of Korea; ^3^ Department of Otorhinolaryngology, Yonsei University College of Medicine, Seoul, Republic of Korea

**Keywords:** alternative splicing, isoform, inner ear, cochlea, hearing, transcriptome diversity

## Abstract

The cochlea is a complex organ comprising diverse cell types with highly specialized morphology and function. Until now, the molecular underpinnings of its specializations have mostly been studied from a transcriptional perspective, but accumulating evidence points to post-transcriptional regulation as a major source of molecular diversity. Alternative splicing is one of the most prevalent and well-characterized post-transcriptional regulatory mechanisms. Many molecules important for hearing, such as cadherin 23 or harmonin, undergo alternative splicing to produce functionally distinct isoforms. Some isoforms are expressed specifically in the cochlea, while some show differential expression across the various cochlear cell types and anatomical regions. Clinical phenotypes that arise from mutations affecting specific splice variants testify to the functional relevance of these isoforms. All these clues point to an essential role for alternative splicing in shaping the unique molecular landscape of the cochlea. Although the regulatory mechanisms controlling alternative splicing in the cochlea are poorly characterized, there are animal models with defective splicing regulators that demonstrate the importance of RNA-binding proteins in maintaining cochlear function and cell survival. Recent technological breakthroughs offer exciting prospects for overcoming some of the long-standing hurdles that have complicated the analysis of alternative splicing in the cochlea. Efforts toward this end will help clarify how the remarkable diversity of the cochlear transcriptome is both established and maintained.

## 1 Introduction

The cochlea is a highly specialized organ responsible for hearing. Its function depends on the intricate organization of its myriad cell types, including mechanosensory hair cells (HCs), more than five types of supporting cells (SCs), and many other non-sensory cells that contribute to the survival and function of the sensory cells. All these constituents of the mature cochlea are generated from the otocyst, a spherical and seemingly homogenous population of epithelial cells. How does the cochlea achieve its distinct anatomy, and how is its remarkable cellular diversity created and maintained? Until now, efforts to address these questions have focused largely on regulation at the transcription level. Conditional knockout studies and transcriptomic analyses have identified key transcription factors governing cochlear development (Elliott et al., 2021). Single cell RNA-sequencing techniques are now revealing transcriptomic heterogeneity with unprecedented resolution (Kelley, 2022). Transcriptional control, however, is only one aspect of genetic regulation. A complete picture of the formation and maintenance of molecular diversity in the cochlea requires consideration of post-transcriptional regulation as well.

Proteomic studies have shown that roughly 37% of human protein-coding genes encode multiple isoforms, allowing the 20,000 genes in the genome to produce more than 290,000 peptides (Kim et al., 2014). Alternative promoters, alternative translation start/stop sites, and post-translational modifications are all examples of post-transcriptional regulatory mechanisms that can contribute to isoform diversity (Hu et al., 2015; Reyes and Huber, 2018). Recent studies have explored the result of these processes in the cochlea, and comprehensive reviews have been written on this subject (Booth et al., 2018a; Shi et al., 2022). In this article, we focus on *alternative splicing* (AS), a post-transcriptional modification process that allows the production of multiple mRNA transcripts from a single gene.

AS is exceptional in both its extent and its contribution to transcriptomic diversity. AS is estimated to occur in roughly 95% of human multi-exon genes (Pan et al., 2008; Wang et al., 2008), about two-thirds of which are tissue-specific (Wang et al., 2008). During development, AS acts as a source of transcriptomic variation required to meet the increasingly specialized demands imposed upon different tissues or cell types (Baralle and Giudice, 2017). Therefore, it is possible that 1) cochlea-specific AS might aid in shaping the unique anatomy of the organ, and 2) region- or cell type-specific AS might help generate heterogeneity within the cochlea.

Although no systematic attempt has yet been made to assess its splicing profiles, several reports hint at an active role for AS in the cochlea. Many of the molecules essential for hearing exist in multiple splice variants, some of which are expressed specifically in the cochlea. Biochemical properties differ among isoforms. Functional and morphological defects observed in isoform-specific knock-out (KO) animals and different clinical phenotypes arising from mutations that affect different isoforms testify to the distinct roles assumed by the splice variants. Furthermore, AS patterns vary according to cell type, spatial location, and developmental stage, suggesting the possibility that AS plays a role in creating morphological and functional heterogeneity within the cochlea. Finally, mice deficient for certain splicing regulators show hearing impairment, although the detailed pathogenic mechanisms underlying this defect remain unclear. These results highlight the importance of AS regulation in the normal development and function of the cochlea.

This article aims to provide a comprehensive review of the literature focusing on the physiological relevance of AS in cochlear development and hearing function. We will first give a basic overview of AS and cochlear biology, and then explore the rich transcriptomic diversity of the cochlea conferred by AS and its regulator RNA-binding proteins (RBPs). Finally, we will discuss clinical cases of hearing loss caused by abnormal AS regulation and potential therapeutic approaches utilizing AS for correcting deafness-causing mutations.

## 2 Background

### 2.1 Overview of alternative splicing

**FIGURE 1:**
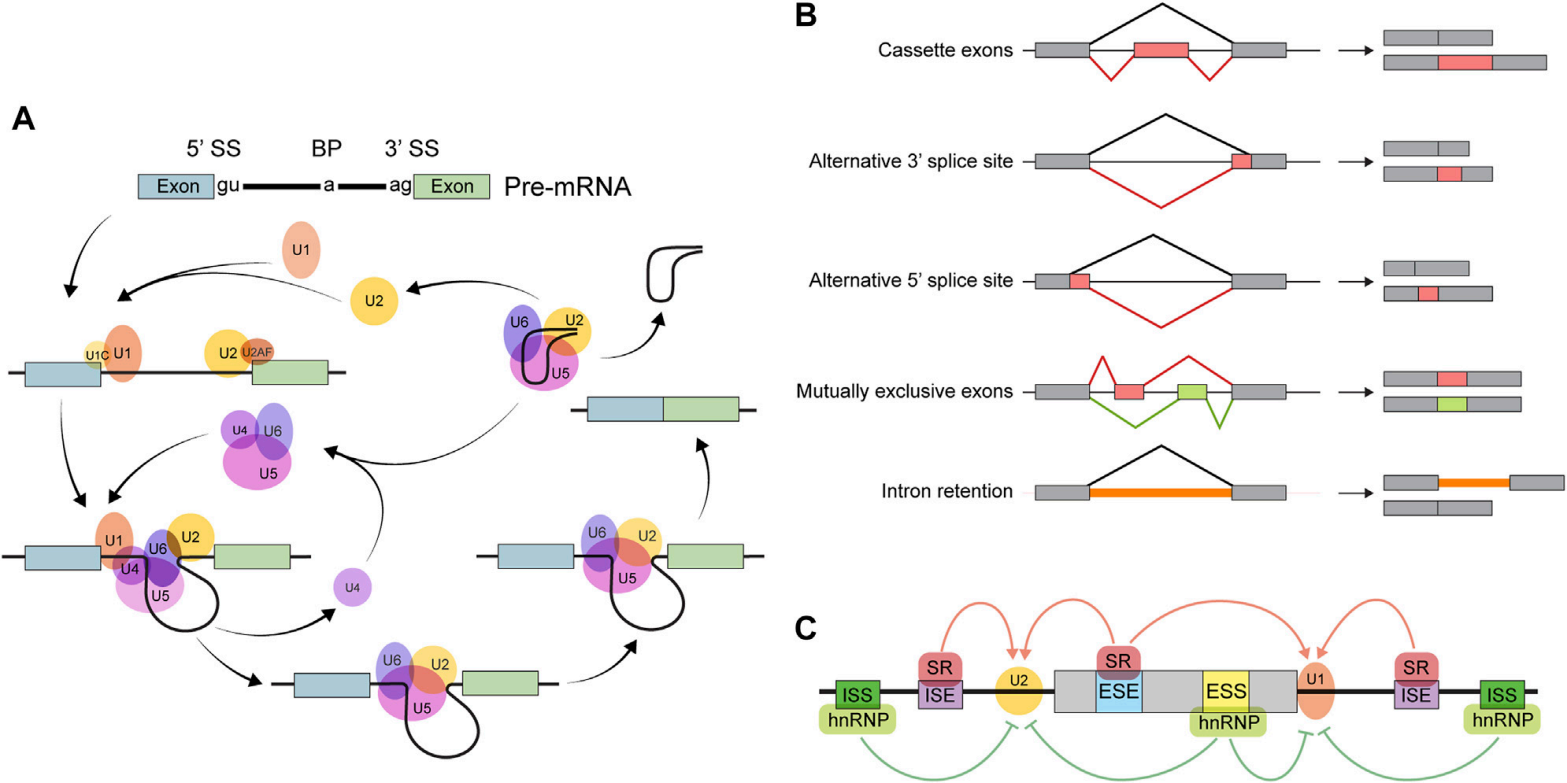
Overview of alternative splicing. **(A)** The splicing reaction. The core spliceosome consists primarily of five small nuclear ribonucleoproteins (snRNPs): U1, U2, U4, U5, and U6. The splicing reaction begins when the U1 snRNP binds the 5′ splice site (5′ SS). Next, U2 and some non-snRNP factors recognize other important sequences, including the 3′ splice site (3′ SS) and the branch point (BP). The resulting RNA-protein complex then undergoes a series of conformational rearrangements, becoming catalytically active. Finally, in a two-step transesterification reaction, two exons are ligated and the intervening intron is spliced out in the form of a ‘lariat’ before being subsequently degraded. **(B)** The major types of binary AS events. **(C)** The determinants of splicing outcomes. Exonic/intronic splicing enhancer (ESE/ISE) sequences are recognized by splicing activators, such as the serine-rich (SR) family of proteins, which facilitates the action of the splicing machinery. Repressors, such as the heterogeneous nuclear ribonucleoproteins (hnRNPs), bind to exonic/intronic splicing silencers (ESSs/ISSs) to inhibit the components of the spliceosome from recognizing splice site sequences.

Primary transcripts from most eukaryotic genes contain *splice sites*. A ribonucleoprotein complex known as the *spliceosome* excises parts of pre-mRNAs whose borders are defined by splice site sequences (Figure 1A); this is how the task of intron removal is accomplished. Some introns are defined by splice sites “strong” enough to be invariably recognized by the spliceosome, so these are constitutively spliced out. Other introns may or may not be removed depending on circumstances. A diverse array of transcripts may be generated from a single gene as a result of such *alternative splicing*. It is customary to describe any AS event as one (or a combination) of 5 types of binary processes: skipping of an alternative or “cassette” exon, alternative 5′ splice site usage, alternative 3′ splice site usage, utilization of mutually exclusive exons, and intron retention (Figure 1B). As a result, the *quantity* and *kind* of processed transcripts are affected. When a premature termination codon is introduced *via* AS, the transcript is likely to be degraded by the non-sense-mediated decay pathway (Titus et al., 2021). Modification of untranslated region (UTR) sequences may influence mRNA localization or stability (Titus et al., 2021). Finally, when a coding sequence is differentially spliced, the resulting protein isoforms may differ in stability, localization, or function (Titus et al., 2021).

Factors affecting splicing outcomes can be broadly categorized as either *cis*- or *trans*-acting (Figure 1C). *Cis*-regulatory elements are pre-mRNA sequences near the splice site that influence recognition by the spliceosome. These are further classified as exonic or intronic splicing enhancers or silencers according to their location and function. *Trans*-regulatory elements refer to factors—usually RBPs—that localize to *cis*-regulatory sequences and affect binding of the splicing machinery to the pre-mRNA. Regulation of *trans*-acting factor expression can alter splicing outcomes to suit various physiologic requirements. In addition, since splicing occurs concurrently with transcription, various aspects of transcription, such as elongation rate, can also affect splicing outcomes (Kornblihtt et al., 2013).

### 2.2 Overview of cochlear structure and function

**FIGURE 2:**
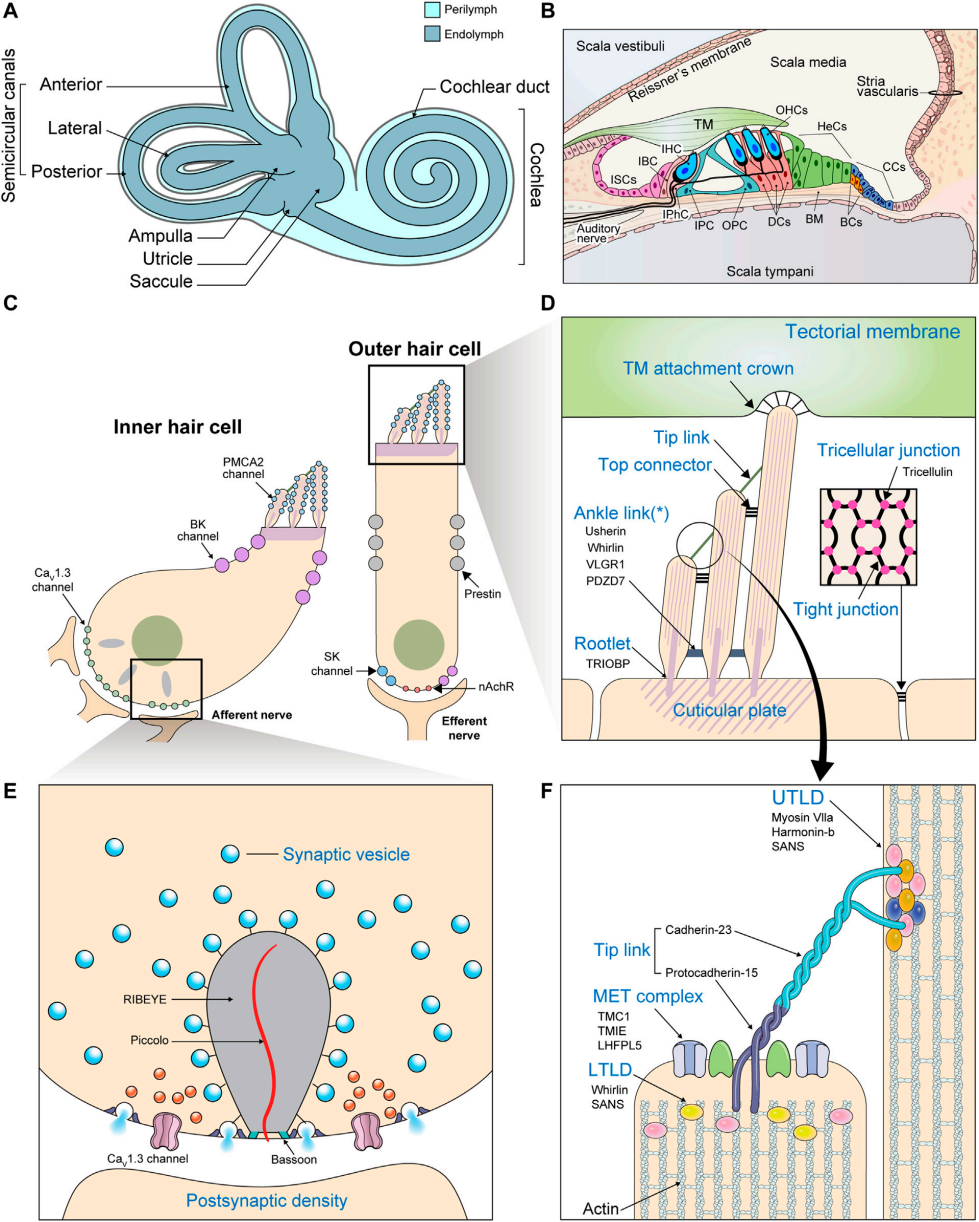
Gross and molecular anatomy of the mammalian cochlea. **(A)** The structure of the inner ear. **(B)** Transverse section of the cochlea. (BC: Boettcher cell; BM: Basilar membrane; CC: Claudius cell; DC: Deiters cell; HeC: Hensen cell; IBC: Inner border cell; IHC: Inner hair cell; IPC: Inner pillar cell; IPhC: Inner phalangeal cell; ISC; Inner sulcus cell; OHC: Outer hair cell; OPC: Outer pillar cell) **(C)** Enlarged view of the two kinds of cochlear HCs depicting the locations of the most important ion channels. Efferent fibers from the olivary complex synapse mainly with OHCs. (BK channel: large-conductance Ca^2+^-activated K^+^ channel; nAChR: α9/α10 nicotinic acetylcholine receptor; SK channel: small-conductance Ca^2+^-activated K^+^ channel) **(D–F)** Important structures are labeled in blue with their molecular constituents listed below in black. **(D)** Enlarged view of the HC apical surface depicting various types of hair bundle links. The asterisk (*) indicates that ankle links are transient structures absent from the mature hair bundle. (Inset: A section parallel to the cochlear floor at the level of the tight junctions. Specialized structures called *tricellular junctions* are formed at points of tricellular contact.) **(E)** The components of a ribbon synapse. **(F)** The components of the tip link and its adjacent structures. The insertion sites of the tip links into neighboring stereocilia appear as electron-dense structures when viewed under electron microscopes, which earned them the names *upper* and *lower tip link densities* (UTLD and LTLD). Various Usher proteins form scaffolds that connect the tip link to the actin core, which is stabilized by other actin-binding proteins.

The cochlea consists of three chambers (Figures 2A,B). The *scala vestibuli* and *scala tympani* are spaces filled with a fluid called *perilymph*. These spaces together function as a conduit for incoming sound waves. Sandwiched between these two spaces is the *scala media*, a blind tube surrounded by three walls. *Reissner’s membrane* and the cochlear *floor* constitute the scala media’s borders with the scala vestibuli and scala tympani, respectively. The wall facing the lateral side houses a special kind of epithelium, the *stria vascularis*, which is important for maintaining ion homeostasis. Vibrations in the perilymph are converted to undulations of the *basilar membrane* underlying the cochlear floor. Within the cochlear floor resides the *organ of Corti*, the auditory sensory organ that harbors the mechanosensory hair cells (HCs) and supporting cells (SCs). Movements of the basilar membrane displace the HCs relative to the overlying *tectorial membrane*, deflecting their *hair bundles*.

Each hair bundle is an array of actin-based protrusions called *stereocilia* located on the apical surface of every HC (Figures 2C,D). A variety of structures work together to translate these mechanical stimulations into electric signals. These include various *hair bundle links*, which ensure the entire hair bundle moves in concert to generate a coherent signal (Figures 2D,F). Horizontal top connectors connect adjacent rows of stereocilia, while tectorial membrane attachment crowns anchor the tips of tallest stereocilia to the tectorial membrane. Some structures, such as *ankle links*, appear transiently during development and are thought to be involved in hair bundle maturation. Most importantly, *tip links* connect the uppermost regions of adjacent rows of stereocilia with one another (Richardson and Petit, 2019). Tip links are somehow coupled to the *mechanoelectrical transduction (MET) machinery*, an apparatus consisting of molecules such as TMC1, LHFPL5, and TMIE that triggers the influx of K^+^ and Ca^2+^ in response to mechanical tension (Zheng and Holt, 2021). Depolarization of the HC membrane leads to additional Ca^2+^ influx through Ca_V_1.3 L-type Ca^2+^ channels, further accelerating depolarization (Brandt et al., 2003; Sidi et al., 2004). Increased intracellular Ca^2+^ concentration leads to K^+^ outflow through Ca^2+^-sensitive K^+^ channels located at the basolateral membranes of the HCs. This triggers cell repolarization so the cycle can begin again (Fettiplace, 2017).

In mammals, the subsequent response depends on the type of HC. Inner hair cells (IHCs) receive the majority of auditory afferent innervation and possess specialized structures called *ribbon synapses* at their interface with nerve fibers (Figures 2C,E). Depolarization in the IHCs leads to glutamate release at these synapses, propagating the sound-evoked electrical signals to the neurons, which carry it to the brain. On the other hand, outer hair cells (OHCs) receive only a limited number of afferent fibers. Electrical excitation of these cells, rather than promoting neurotransmission, triggers cell contraction (Fettiplace, 2017). This *cochlear amplifier* mechanism acts to augment the acoustic signal in a non-linear fashion.

## 3 Cochlear transcriptomic diversity from alternative splicing

The intricate organization and function of the cochlea suggests its transcriptome must also be quite specialized and diversified. Does AS contribute to sculpting the unique and diverse transcriptome of the cochlea? Compiling known cases of cochlear AS could be a good starting point for answering this question, but few such attempts have been made. One study found 20 unannotated, highly conserved exons in 12 deafness-related genes (Ranum et al., 2019). This hints that vast areas of the cochlear transcriptome remain unexplored. Although unbiased, transcriptome-wide analyses are still wanting, some isoforms of important proteins have been documented. For example, proteins related to *Usher syndrome*—a group of genetic conditions characterized by hearing loss and blindness—tend to exist in multiple isoforms with unique functions and distribution patterns (Whatley et al., 2020). An earlier review summarized the most well-known examples of cochlear AS (Wang et al., 2016). Here, we have created a more comprehensive and up-to-date catalogue of cochlear splice variants, which is presented in Supplementary Table S1.

In this section, we will examine whether and how AS helps to address the following two challenges: to specialize the cochlear transcriptome so that it becomes distinct from that of other organs, and to diversify the transcriptome so as to distinguish various cochlear cell types or anatomical regions. One caveat is that transcript diversity does not necessarily translate into diversity at the protein level. And even when it does, the mere existence of protein isoforms is not necessarily evidence of functional relevance. Ideally, we need clinical or experimental evidence that depletion of different isoforms affects cochlear anatomy or physiology in different ways. Biochemical studies could directly prove that molecular characteristics differ among isoforms, but it is possible that each splice variant performs its unique function in every cell, whatever the circumstances. To argue that AS accounts for differences among cells or tissues, it must be shown that the expression of the splice variants is differentially regulated in those environments. With this in mind, we now proceed to inspect several selected AS events and discuss their potential significance.

### 3.1 Alternative splicing events with cochlea-specific roles

How does the cochlear transcriptome differ from that of other organs, and how does AS contribute to this difference? To date, no high-throughput study has yet been conducted specifically to address this question, but the wealth of publicly available RNA-sequencing datasets provides opportunities to mine cochlea-specific AS events. One study took advantage of published transcriptome datasets to compare exon inclusion rates in different cell types (Ling et al., 2020). Although the focus was on neurons, the paper presented as an example one cassette exon of *Sptan1* used specifically in cochlear HCs. *Sptan1* is a cytoskeletal protein expressed broadly in many tissues, and has been shown *in vivo* to be necessary for hair bundle morphogenesis and HC survival (Yao et al., 2022). It would be worth investigating whether the exon confers an HC-specific function to the protein.

Further research using a similar approach will help to characterize the unique cochlear transcriptome on a large scale. At present, a number of studies have reported inner ear- or cochlea-specific AS in genes important for hearing. We will examine those examples and try to infer the role of such AS events in cochlear function.

#### 3.1.1 Cadherin 23

**FIGURE 3:**
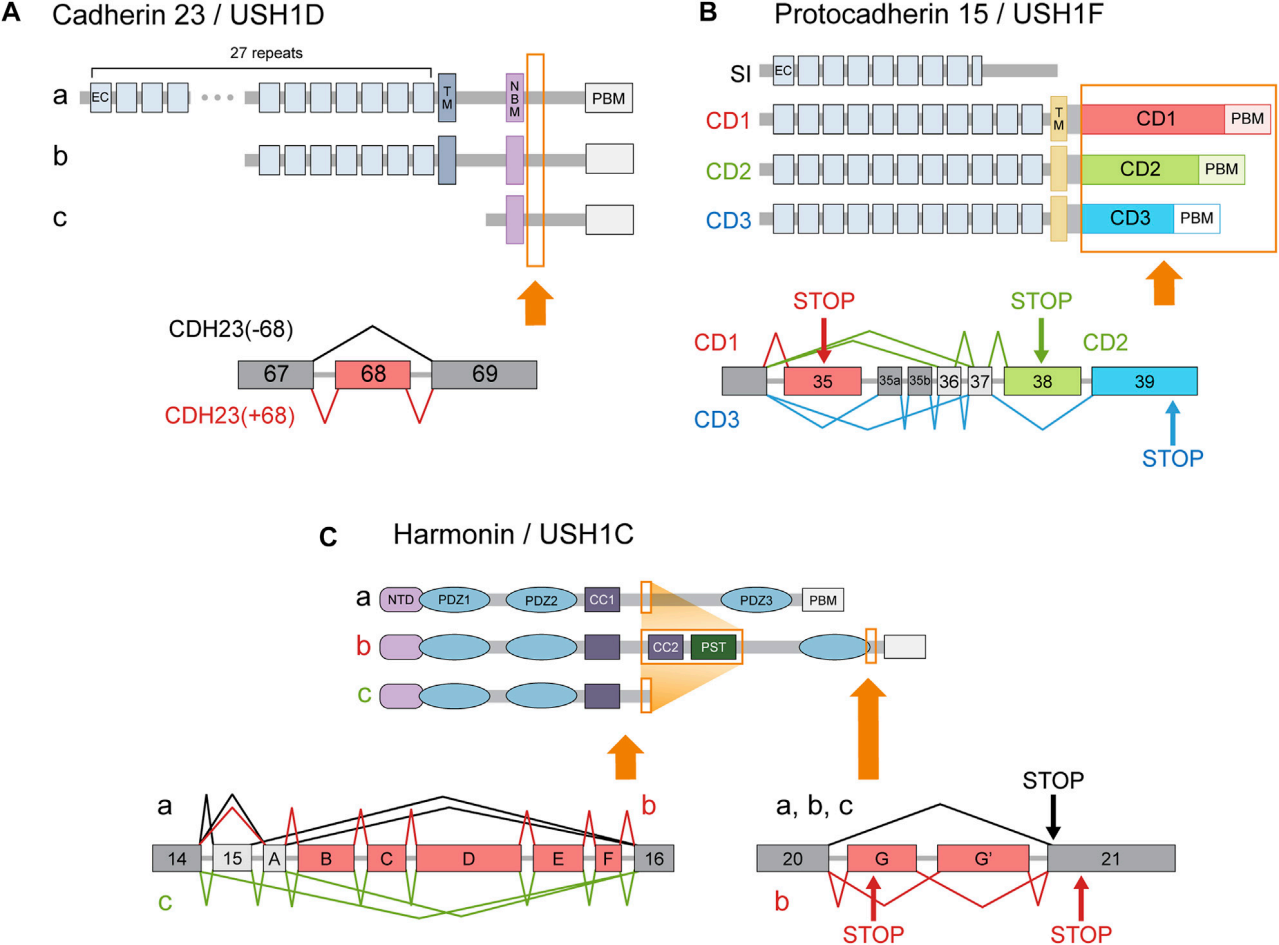
Usher gene splice variants. The major classes of Usher protein isoforms and the AS events responsible for producing them. Protein regions affected by AS are indicated in orange boxes. In the transcript diagrams, the different colors indicate different splice variant classes. Exons (or more generally, transcript regions) selectively utilized in a certain class are indicated with corresponding colors. Dark gray boxes represent constitutively included exons, while light gray boxes denote alternative exons that do not belong to a single isoform class. Arrows labeled ‘STOP’ indicate stop codons specific to the splice variant class of the corresponding color. **(A)** Cadherin 23 isoform classes a, b, c are produced by utilization of alternative promoters. All three types of transcripts can either include or exclude exon 68. **(B)** Splice variants of protocadherin 15 are classified into 4 categories according to their cytoplasmic domains. Although AS can also affect the number of EC repeats, 11 repeats are usually assumed to comprise the standard extracellular domain. **(C)** Splice variants of harmonin are divided into three classes—a, b, and c—according to the arrangement of the functional domains in their encoded proteins. (CC: coiled coil domain; CD: cytoplasmic domain; EC: extracellular cadherin repeat; NBM: harmonin N-terminal-binding domain; NTD: N-terminal domain; PBM: PDZ-binding motif; PST: proline, serine, threonine-rich region; TM: transmembrane domain).

Cadherin 23 (CDH23, USH1D) is an Usher protein that constitutes the upper part of the stereociliary tip link (Figure 2F). The inclusion of *CDH23* exon 68 is probably the best-known inner ear-specific AS event (Figure 3A) (Di Palma et al., 2001; Siemens et al., 2002; Siemens et al., 2004; Xu et al., 2008). Given the essential role of CDH23 in hearing, the specificity of this AS event strongly suggests the peptide encoded by exon 68 (hereafter abbreviated peptide 68) must perform some function unique to the inner ear. For example, one study reported that peptide 68 contained a cysteine residue that could induce conformational changes in a redox-dependent manner. The authors speculated that this may help relieve some of the mechanical stress caused by stereociliary deflection (Yonezawa et al., 2008). But since the cytoplasmic domain of CDH23 has been best characterized in its interaction with other Usher proteins such as harmonin (USH1C), most efforts to determine the significance of peptide 68 have focused on these interactions as well.

CDH23 interacts with a PDZ domain of harmonin *via* its C-terminal PDZ-binding motif (PBM) (Boëda et al., 2002; Pan et al., 2009), and with an N-terminal domain (NTD) of harmonin *via* an internal cytoplasmic domain (Siemens et al., 2002; Pan et al., 2009). *In vitro* comparisons of variants with and without peptide 68 have generally indicated that peptide 68 inhibits the CDH23-harmonin interaction (Siemens et al., 2002; Xu et al., 2008; Xu et al., 2010). One group conjectured that harmonin may not be the primary interaction partner of CDH23, and used yeast two-hybrid screening to identify two novel PDZ domain-containing proteins that bind CDH23: MAGI-1 and PIST (Xu et al., 2008; Xu et al., 2010). Harmonin, MAGI-1, and PIST compete for the same C-terminal PBM on CDH23. The presence of peptide 68 induced stronger colocalization of CDH23 with PIST than with harmonin, leading to its partial retention in the trans-golgi network (Xu et al., 2010).

In contrast, a separate study reported that peptide 68 is a facilitator of protein-protein interactions. Wu et al. (2012) found that peptide 68 can bind two mutually exclusive targets: either the harmonin NTD or peptide 68 of another CDH23 molecule. Large-molecular weight complexes formed when CDH23 and harmonin were mixed, but only in the presence of peptide 68. Peptide 68 seemed, therefore, to contribute to CDH23-harmonin complex formation by providing an additional interface for harmonin NTD-binding and by facilitating self-dimerization. The authors speculated that such polymers could create a dense web-like structure that augments the stability of the stereociliary tip links (Wu et al., 2012). This hypothesis would explain the significance of inner ear-specific AS of *CDH23*, but it will have to be corroborated with biophysical measurements. To pinpoint the exact role of peptide 68 in cochlear function, it will be necessary to study exon-specific KO models.

#### 3.1.2 Harmonin

Another well-known inner ear-specific AS event concerns harmonin (USH1C), a scaffolding protein thought to act as a hub for Usher protein interactions (Reiners et al., 2006; Whatley et al., 2020). Harmonin splice variants comprise three classes: a, b, and c (Figure 3C). Class a includes the canonical isoform, as well as an alternative transcript that utilizes exon A instead of exon 15. Class a proteins possess three PDZ domains. Class b includes transcripts that lack exon 15 but contain alternative exons A-F, which encode additional functional domains. Class c, which includes the remaining isoforms, encodes proteins with only two PDZ domains (Verpy et al., 2000).

Harmonin-b is considered the primary isoform in the cochlea. Two types of class b transcripts are detected only in the inner ear (Verpy et al., 2000), and harmonin-b proteins are restricted to HC stereocilia (Figure 2F) (Boëda et al., 2002; Michalski et al., 2009). Whereas harmonin-a and c mainly interact with other Usher proteins, the domains encoded by the alternative exons allow harmonin-b to interact with actin filaments and harmonin molecules as well (Boëda et al., 2002; Adato et al., 2005). Mice homozygous for a mutation that specifically affected the b isoform were profoundly deaf and had vestibular dysfunction (Johnson et al., 2003), which is probably caused by the structural and functional defects in OHCs and the ensuing HC degeneration (Johnson et al., 2003; Michalski et al., 2009). In contrast, harmonin-a is expressed broadly, including in the retina (Verpy et al., 2000). Harmonin-a proteins localize to the basolateral membranes of retinal photoreceptors (Reiners et al., 2003) and HCs (Gregory et al., 2011), rather than to their apical surfaces or stereocilia. Class a isoforms associate with Ca_V_1.3 channels, which reduces the number of channels available at the basal cell surface (Gregory et al., 2011).

These findings suggest a model in which the more widespread harmonin-a performs some common function in regulating synaptic transmission at the basal membranes of retinal photoreceptors and inner ear HCs, while the inner ear-specific harmonin-b uses its additional functional domains to target apical plasma membranes and form scaffolds therein. Clinical data contribute to the plausibility of this model. Mutations in *USH1C* exons B and D underlie DFNB18, a non-syndromic hearing loss disorder (Ouyang et al., 2002). In addition, a mutation in exon 15 was identified as the pathological variant in some families with retinitis pigmentosa, which is the ocular component of the Usher phenotype (Khateb et al., 2012). Patients with this variant displayed late-onset, mild-to-severe hearing impairment (Khateb et al., 2012). Hearing loss due to *USH1C* mutation is usually congenital and profound (Castiglione and Möller, 2022), so the newfound mutation represented the first deviation from this standard pattern (Khateb et al., 2012). These observations make sense when isoform distribution is considered; mutations in inner ear-specific exons should spare the retina and exon 15 mutations should still allow proper class b isoform function in HC stereocilia. Therefore, organ-specific AS is essential for harmonin’s unique localization and scaffolding function in the inner ear.

#### 3.1.3 Protocadherin 15

Protocadherin 15 (PCDH15, USH1F) is the component of the stereociliary tip link that connects to both CDH23 and the MET machinery (Figure 2F). Many PCDH15 splice variants exist in the cochlea, with transmembrane isoforms belonging to one of three categories—CD1, CD2, and CD3—according to their cytoplasmic domains (Figure 3B) (Ahmed et al., 2006; Ahmed et al., 2008). *USH1F* mutations usually manifest with hearing loss accompanied by vestibular and visual defects, but mutations that selectively affect CD2 isoforms cause non-syndromic hearing loss (Pepermans et al., 2014). This suggests CD2 splice variants perform some unique function in the mature cochlea. Unlike in our previous examples, however, this organ-specificity cannot be attributed to differential AS alone. Although it is true that the distribution of the CD1 and CD2 isoforms is restricted compared to the CD3 isoforms, CD2 isoforms appear in several tissues other than the cochlea, such as the retina, spleen, and testis (Ahmed et al., 2008).

The uniqueness of the cochlea in this case seems to lie in its specific requirement for AS, rather than in its AS profile. While each isoform is dispensable for the initial formation of hair bundles and for acquisition of MET (Webb et al., 2011), CD2 isoforms are necessary for maintenance of the tip links and for hearing at later stages (Webb et al., 2011; Pepermans et al., 2014). We can speculate as to the molecular nature of this isoform-specific requirement. The subcellular expression patterns of the three isoform classes are distinct and change dynamically during development (Ahmed et al., 2006; Michel et al., 2020). Perhaps, the precise localization of each isoform is essential in the mature cochlea but not in the vestibule, retina, or immature cochlea. It is also possible that PCDH15-CD2 binds directly to the MET machinery component TMIE, whereas the interactions of the CD1 and CD3 isoforms with TMIE depend on LHFPL5 (Zhao et al., 2014). The CD2 isoforms might be required because of the relatively low level of LHFPL5 expression in the mature cochlea (Xiong et al., 2012; Mahendrasingam et al., 2017) or because of some unique geometric configuration formed by the direct interaction between PCDH15-CD2 and TMIE. Regardless, the example of PCDH15 illustrates that unique requirements for AS can also contribute to the specialization of the cochlear transcriptome.

### 3.2 Cell type-specific splicing in the cochlea

Although the cochlea is a small organ, it is astoundingly complex. It is conceivable that AS might help achieve the precise level of transcriptomic heterogeneity required for creating distinct anatomic regions and cell types within the cochlea. One study using single cell long-read RNA-sequencing found considerable AS variation between cell types, although the authors only provided a detailed analysis of isoform diversity and distribution for one example gene (Ranum et al., 2019). Further unbiased analyses of cell type-specific splicing profiles are needed to bolster this hypothesis.

**FIGURE 4:**
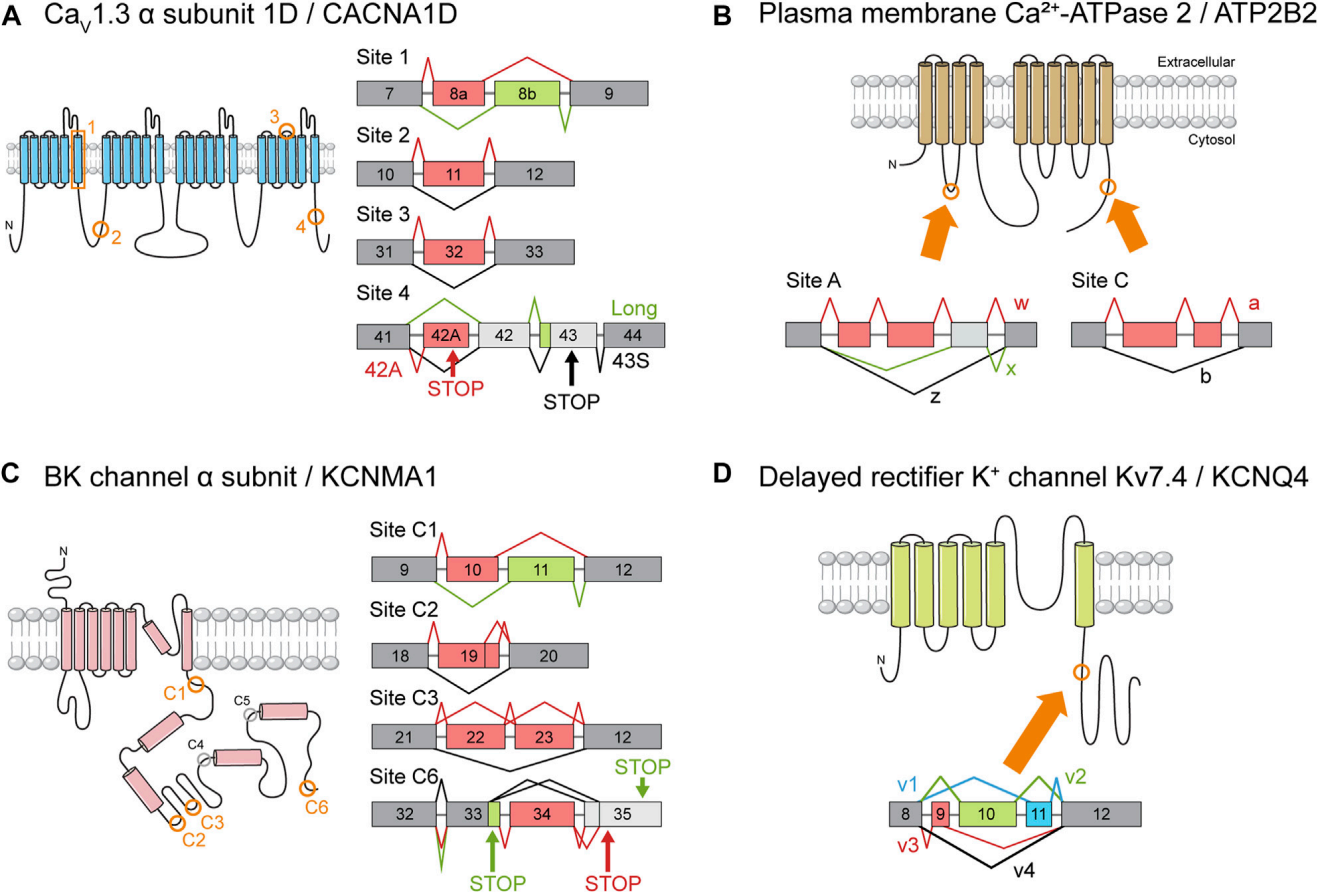
Ion channel gene splice variants. The structure of ion channel proteins and important AS events. The protein regions affected by AS of each channel are indicated by orange boxes or circles. The meaning of the color schemes is the same as in Figure 3. **(A)** Splice variants of Ca_V_1.3 α subunit 1D (CACNA1D). Splice site numbering is arbitrary. Only some of the reported AS events are depicted. Please refer to Table S1. **(B)** Splice variants of plasma membrane Ca^2+^-ATPase 2 (PMCA2). **(C)** Splice variants of the pore-forming BK channel α subunit (KCNMA1). Cytoplasmic splice sites are circled, and the sites reported to be utilized in murine inner ears by Miranda-Rottmann et al*.* (2010) are colored in orange. **(D)** Splice variants of delayed rectifier K^+^ channel Kv7.4 (KCNQ4) as identified by Beisel et al. (2005).

There are only a few published reports of cell type-specific splicing, most of which relate to genes encoding ion channels. C-terminal splice variants of *CACNA1D* encode truncated Ca_V_1.3 channel α subunits with peculiar electrophysiological characteristics (Figure 4A). One isoform that is expressed more abundantly in OHCs than in IHCs nearly lacks the Ca^2+^-dependent inactivation property typical of Ca_V_1.3 channels. It is possible that current through the channels may drive synaptic transmission to the few afferent nerve endings that terminate at the OHCs, mediating activity-dependent transcriptional changes during maturation or in some way contributing to the cochlear amplifier function (Shen et al., 2006).

Another example is the *ATP2B2* gene, which encodes the PMCA2 channels that are the primary route by which Ca^2+^ is removed from the HCs (Lumpkin and Hudspeth, 1998). Two classes of splice isoforms, w and z, differ in the number of cassette exons inserted in a specific splice site (Figure 4B). As a result, w and z isoforms are selectively targeted to the apical and basolateral membranes of epithelial cells, respectively (Chicka and Strehler, 2003; Grati et al., 2006; Hill et al., 2006). The w isoform is predominant in cochlear HCs, where the channels are localized exclusively to stereociliary hair bundles (Grati et al., 2006; Hill et al., 2006; Chen et al., 2011). In contrast, the z isoform is expressed mainly in spiral ganglion neurons where it localizes to the membranes of their cell bodies and neurites (Chen et al., 2011). It is plausible that AS serves to optimize PMCA2 function according to the unique Ca^2+^ dynamics of each cell type. The validation of these hypotheses will require cell type-specific knockout studies.

A final example illustrates how cell type-specific transcriptome data could be exploited to analyze differences in AS patterns. In a feat of precision, He et al. (2000) pioneered the suction pipette technique, in which dissociated cells are manually separated based on morphological differences (He et al., 2000). Transcriptomes of 4 cell types have been characterized using this method (Liu et al., 2018). Zhou et al. (2021) probed this database for previously unknown splice variants of MET machinery components. Four AS events were identified in the *Tmc1*, *Lhfpl5*, and *Tmie* genes and subsequently verified through RT-PCR. Three of these events occurred in significantly higher frequencies in OHCs than in IHCs (Zhou et al., 2021). Although they avoided speculation as to the possible implications of these data, the authors of the study did demonstrate that data generated *via* high-throughput pipelines can help identify potentially important AS events.

### 3.3 Alternative splicing and cochlear tonotopy

**FIGURE 5:**
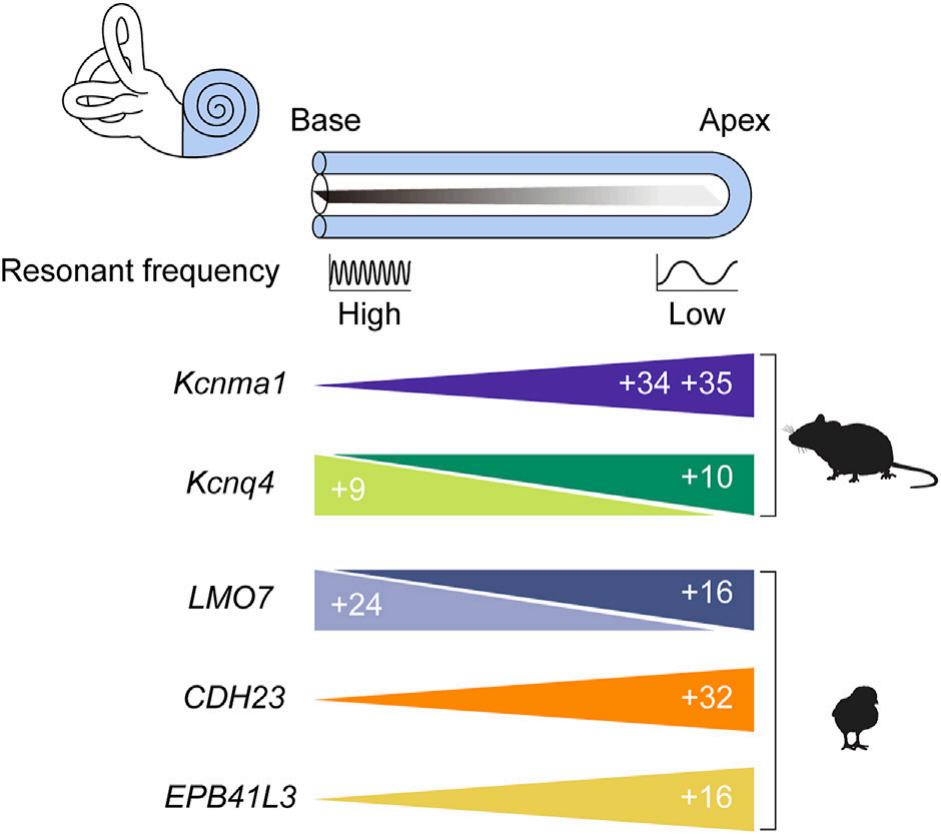
Tonotopic AS events. The cochlea is depicted unwound such that its longitudinal axis is straightened. The region of the straightened cochlear duct farthest from the middle ear is called the *apex*, the most proximal region is the *base*, and the virtual line joining these regions is the *longitudinal* or *tonotopic axis*. The colored triangles represent the relative abundance of splice variants for the indicated gene along the axis. The numbers inside the triangles refer to the exons that characterize each variant, and the cartoons next to the triangles indicate the organism in which the tonotopic expression of each splice variant has been verified.

We now turn to a particularly interesting case of heterogeneity within the cochlea, that of tonotopy. The information content of a sound is encoded in its frequency makeup. How does the auditory system analyze and interpret this signal? Viewed from the middle ear, the distal end of the cochlear duct is called the *apex*, whereas the most proximal region is called the *base* (Figure 5). Various mechanical and electrophysiological parameters vary systematically along the apicobasal (longitudinal) axis. As a result, the *resonant frequency* of each HC—the input frequency at which its receptor potential amplitude is maximized—exhibits a spatial gradient. The apex of the cochlea responds most efficiently to low-frequency sounds, whereas the base responds best to high-frequency sounds. Taking advantage of this organization, which is known as *tonotopy*, the cochlea effectively performs a Fourier transformation, such that the frequency composition of the sound stimulus is converted into an amplitude distribution of HC receptor potentials along the longitudinal axis (Fettiplace, 2020).

Many studies have tried to uncover the molecular basis of tonotopy (Frucht et al., 2011; Kowalik and Hudspeth, 2011; Son et al., 2012). Although there has been remarkable progress in elucidating the formation and maintenance of tonotopy during cochlear development (Mann et al., 2014; Thiede et al., 2014; Son et al., 2015; Koo et al., 2023), the molecular apparatus responsible for transforming an embryonic blueprint into an actual gradient of anatomical and physiological features is largely unknown. Here, we explore those studies that included AS in their attempts to solve the riddle of tonotopy. Overall, the results are far from conclusive, perhaps again owing to the technical difficulties of having to analyze subregions of an already miniscule organ. Nevertheless, these offer tantalizing hints towards a plausible hypothesis that AS complements the regulation of gene expression required to create spatial heterogeneity within the cochlea.

#### 3.3.1 BK channels

In amphibians and reptiles, a short tubular structure called the *basilar papilla* takes on the role of sound detection. Unlike in the cochlea, where gradients in the mechanical properties of the basilar membrane are most relevant for frequency discrimination, it is *electrical resonance* that predominates in the basilar papilla. HC resonant frequency is determined by the properties of each cell’s ion channels (Fettiplace, 2020). In particular, the density and kinetics of large-conductance Ca^2+^-activated K^+^ channels, or BK channels, are the major determinants of resonant frequency in turtles (Crawford and Fettiplace, 1981; Art and Fettiplace, 1987; Art et al., 1995; Wu et al., 1995). These are the channels primarily responsible for the fast outward K^+^ currents observed in IHCs.

Channel density might be controlled through the regulation of gene expression, but how might the kinetic properties of a channel be tweaked to form a systematic gradient? It was hypothesized that AS of *KCNMA1*, the gene encoding the pore-forming α subunit, underlies this phenomenon. Two studies examined the chicken basilar papilla and found isoforms with distinct expression patterns along the longitudinal axis also showed distinct electrophysiological properties (Navaratnam et al., 1997; Rosenblatt et al., 1997). Studies in turtles yielded similar results (Jones et al., 1998; Jones et al., 1999a; Jones et al., 1999b). Models incorporating splice variant distribution succeeded in explaining a frequency range of 300–1,100 Hz (Ramanathan et al., 2000; Ramanathan and Fuchs, 2002). Although this range fell short of covering the entire range of chicken hearing (up to roughly 5,000 Hz), there was reason for optimism. Perhaps unknown splice variants or subunits account for the unexplained portion of the hearing range (Ramanathan and Fuchs, 2002). After all, the combinatorial inclusion of 11 alternative exons at 7 splice sites could in theory generate up to 576 splice variants (Navaratnam et al., 1997; Rosenblatt et al., 1997). The fact that only a few transcripts have been detected (Ramanathan et al., 2000) may indicate the existence of undetected isoforms.

Later investigations employing genome-wide technologies, however, yielded results that were far from what was hoped for (Miranda-Rottmann et al., 2010). The transcript diversity promised by AS failed to materialize, with only 4 of the 7 potential AS sites being utilized (Figure 4C). This meant that the number of possible combinations fell from 576 to only 48. Worse still, only 28 of those 48 were detected in the basilar papilla. Then, only 5% of the identified transcripts included alternative exons. Even after compensating for possible artifacts, the estimated proportion of transcripts undergoing AS was no more than 30%. No systematic differences in exon combinations were observed between the basal and apical regions. Transcripts containing exons 34 and 35, which encode slower channels, tended to be more abundant in the apex (Figure 5), but the difference between fast and slow channels seemed too meager to explain the observed variance in kinetics (Miranda-Rottmann et al., 2010). In summary, the contribution of AS to the variation of BK channel kinetics seems minor if it exists at all. There have been attempts to interpret the significance of *KCNMA1* splicing in different contexts (Kim et al., 2010; Sakai et al., 2011), but the gradient of AS that does exist along the tonotopic axis still awaits interpretation.

#### 3.3.2 Kv7.4

The differential AS of an ion channel gene along the tonotopic axis has been pointed to as the explanation for some peculiar characteristics of a congenital hearing loss disorder. The voltage-gated K^+^ channel Kv7.4, which is known as a delayed rectifier potassium channel for its function in cardiomyocytes, is encoded by the *KCNQ4* gene. This channel accounts for the majority of outward K^+^ conductance in mature OHCs, although IHCs also partly depend on this channel (Oliver et al., 2003). Thus, it is unsurprising that those who mapped the *KCNQ4* gene to the autosomal-dominant deafness locus DFNA2 immediately pointed to OHC dysfunction as the key pathological mechanism (Kubisch et al., 1999). Their observation that *KCNQ4* transcripts are expressed specifically in OHCs lent further credibility to their hypothesis (Kubisch et al., 1999).

One problem with their hypothesis, however, was that the eventual degree of hearing loss was more severe than what would be expected from OHC dysfunction alone (Marres et al., 1997). To clarify the reason for this discrepancy, Beisel et al. (2000) examined *Kcnq4* expression in the mouse cochlea, finding the highest levels of expression in basal auditory neurons and IHCs, as well as a reciprocal gradient in OHCs (Beisel et al., 2000). Combined with the slow progression of the relevant deafness from high to low frequencies (Marres et al., 1997), it seemed that spiral ganglion neuron or IHC pathology may be to blame for the progressive hearing loss of DFNA2. Studies using mouse models, however, showed that *Kcnq4* disruption selectively affected OHCs, leaving IHC morphology and function mostly intact. Of note, OHC degeneration progressed from the base towards the apex (Kharkovets et al., 2006), implying that *Kcnq4* deficiency affects more severely the regions in which its expression levels are normally the lowest.

Although this puzzle is far from solved, studies of *Kcnq4* AS might provide some hints. Four splice isoforms of *Kcnq4*, v1-4, have been identified (Figure 4D), and the v3 variant was predominant in both types of HCs in the basal region (Figure 5) (Beisel et al., 2005). In addition to affecting channel expression levels, AS affected the electrophysiological properties of each variant as well (Xu et al., 2007). Perhaps OHC survival depends on the base-specific *Kcnq4* variant v3 rather than on overall gene expression. Investigating such possibilities may help us uncover the roles played by the tonotopic AS of *Kcnq4* in cochlear physiology. For example, why is there a gradient in the size of the outward K^+^ current along the tonotopic axis (Jeng et al., 2020)? As with the case of *KCNMA1*, the phenomenon is there, but its significance needs to be elucidated.

#### 3.3.3 Transcriptome-wide studies of tonotopic splicing

With the considerable interest surrounding tonotopy, it is surprising how little attention has been given to tonotopic AS. Although many studies have used transcriptome-wide techniques to identify tonotopically expressed genes (Frucht et al., 2011; Kowalik and Hudspeth, 2011; Son et al., 2012), only one study examined splicing differences. Koo et al. (2021) divided the chicken basilar papilla at post-hatch day 1 into three segments along the longitudinal axis and looked for differential inclusion of cassette exons (Koo et al., 2021). Genes such as *LMO7*, *EPB41L3*, and *KCNMA1* showed tonotopically differential AS, and this was confirmed *via* RT-PCR (Figure 5). One specific C-terminal tail variant of *KCNMA1* was more abundant at the apex than either the middle or the base, which was consistent with previous isoform distribution measurements (Rosenblatt et al., 1997; Miranda-Rottmann et al., 2010). In another example validated by RT-PCR, *CDH23* exon 32, the chicken exon orthologous to mouse *Cdh23* exon 68, showed differential inclusion in a gradient increasing from base to apex (Koo et al., 2021). This novel result requires further verification, but if it is true, it suggests several interesting questions. For example, how is the tonotopic splicing of *CDH23* related to the gradient of hair bundle mechanical properties observed along the longitudinal axis (Tobin et al., 2019)? Overall, this study illustrates the strengths of an unbiased approach towards AS analysis: past findings are readily verified, novel events are easily discovered, and sometimes unexpected heterogeneity may emerge from well-known results.

## 4 Regulation of alternative splicing in the cochlea

The discoveries outlined above offer a glimpse into the rich diversity of the cochlear transcriptome created by AS. But which *trans*-acting regulators are responsible for those AS events? Research in this direction has only just begun. For example, two studies attempted to uncover the basis of the tonotopic gradient of AS we introduced in the previous section (Miranda-Rottmann et al., 2010; Koo et al., 2021). One study conducted a microarray analysis of the chicken basilar papilla and found no significant difference in the expression of splicing regulators between the apex and the base, but quantitative PCR results indicated that some regulator transcripts have been filtered out of the microarray results (Miranda-Rottmann et al., 2010). Using RNA-sequencing, another study identified tonotopic expression of several splicing regulator genes, including *PTBP3*, *ESRP1*, and *ESRP2* (Koo et al., 2021). Interestingly, by performing a motif enrichment analysis, the authors also found an enrichment of PTB-like binding motifs and ESRP motifs near alternative exons (Koo et al., 2021). These could be taken as indirect evidence of the importance of RBPs in forming the tonotopic organization of the cochlea.

To infer causality, however, knockout experiments would have to be conducted. Only a handful of such endeavors have been undertaken, but even the results of such loss-of-function analyses should be interpreted with caution. Because constitutive splicing is obviously necessary for cell survival, any abnormal phenotype may simply indicate a requirement for that RBP’s role in constitutive splicing. Specific expression must be confirmed to argue for the importance of an RBP in regulating AS. Also confounding any conclusion are the many roles RBPs play in mRNA regulation, which include coordination of translation, monitoring of mRNA stability and decay, and mRNA transport between organelles (Gerstberger et al., 2014). Indeed, there is ample evidence that RBPs regulate cochlear development *via* mechanisms unrelated to AS (Shi et al., 2022). Therefore, the effect of an RBP on cell function or survival cannot be attributed to AS alone without sufficient evidence. All told, not a single RBP has been conclusively proven to govern the unique AS events of the cochlea. The diverse array of splice variants introduced thus far will require much more work to explain.

### 4.1 ESRP1

The only splicing regulator that may legitimately be considered necessary for cochlear development is ESRP1. In the fibroblast growth factor (FGF) signaling pathway, three FGF receptors (FGFRs) are alternatively spliced to produce two isoforms each. Selective expression of these isoforms enables specific communication across the epithelial-mesenchymal boundary (Zhang et al., 2006). ESRP1 was initially identified in a screen for RBPs governing the production of epithelium-specific FGFR isoforms (Warzecha et al., 2009). Since FGF signaling is implicated in various stages of cochlear development (Ebeid and Huh, 2017), the identification of *ESRP1* mutations in a patient with congenital hearing loss naturally led one group of researchers to hypothesize that ESRP1-mediated splicing of FGFRs is essential for hearing (Rohacek et al., 2017). Indeed, mice deficient for *Esrp1* displayed a widened and shortened cochlear duct, expanded Reissner’s membrane, and a contracted stria vascularis. A sequencing analysis of the mutant embryos revealed expression of the mesenchymal FGFR2-IIIc isoform in the cochlear epithelium. Unlike the typical epithelial FGFR2-IIIb isoform, which binds the ligand FGF10, FGFR2-IIIc binds FGF9 and induces inappropriate FGF signaling. Reduction of FGF9 gene dosage restored the proportions of Reissner’s membrane and the stria vascularis almost to normal, proving that the abnormalities in non-sensory development were indeed attributable to aberrant FGFR splicing (Rohacek et al., 2017).

The case of ESRP1 clearly illustrates that AS fine tunes developmental signaling to enable precise communication between tissue compartments. In this case, however, the RBP itself is broadly expressed in epithelial tissues and governs an epithelium-specific splicing program rather than performing an organ-specific function. In fact, *Esrp1* mutant mice exhibit many phenotypes other than inner ear malformation, including cleft lip and palate arising from a defective epithelial-to-mesenchymal transition (Lee et al., 2020). To identify the regulators that shape the unique splicing landscape of the cochlea, it will perhaps be more prudent to take inspiration from mutants that display more specific phenotypes. This is what we will do in the following paragraphs.

### 4.2 SRRM4

The *Bronx Waltzer* (*bv*) mouse, a spontaneous mutant characterized by recessively inherited hearing and balance abnormalities, harbors a large deletion in *Srrm4* (*nSR100*). *Srrm4* encodes a splicing factor predominantly expressed in neurons and HCs (Nakano et al., 2012). A microarray analysis identified several AS defects in the vestibular organs of *bv* mice, including an aberrant skipping of *Rest* exon 4 (Nakano et al., 2012). REST was a transcription factor described *in vitro* as a target of SRRM4. SRRM4 promoted *Rest* exon 4 inclusion, which reduced REST’s transcriptional repressor activity, upregulating expression of its target genes (Raj et al., 2011). Experiments in an exon-specific KO mouse model showed that *Rest* exon 4 was indeed necessary for HC survival (Nakano et al., 2018). Later, the REST-dependence of the *Srrm4*-induced cochlear phenotype was confirmed when transgenic expression of a dominant-negative REST restored HC numbers and hearing function to *bv* mice (Nakano et al., 2020). This was the first splicing regulator that was found essential for hearing, and it remains, alongside ESRP1, one of the only examples in which AS has been conclusively demonstrated to be the underlying mechanism.

The *bv* mutation has long been noted to affect IHCs more severely than OHCs (Whitlon et al., 1996; Sobkowicz et al., 2002). SRRM4 is expressed in both IHCs and OHCs in wildtype mice, and absent from both cell types in *bv* mice (Nakano et al., 2012). What difference between the two types of HCs could account for this phenotypic discrepancy? It was later found that OHCs transiently repress *Rest* transcription independently of SRRM4 during development (Nakano et al., 2020). The authors proposed a model that includes a negative feedback loop between REST and SRRM3, a splicing regulator expressed in HCs that inactivates REST like SRRM4. Once SRRM3 is derepressed in OHCs due to transient REST downregulation, it inactivates REST independently of SRRM4 and ensures cell survival (Nakano et al., 2020). The precise relationship between SRRM4, REST, and SRRM3 activity and cell survival remains unclear. Mice doubly deficient for *Srrm3* and *Srrm4* suffer degeneration of both IHCs and OHCs, although this could only be confirmed in culture conditions due to the early lethality of the double mutant. Forced expression of dominant-negative REST restored survival to IHCs but not OHCs (Nakano et al., 2020). These results suggest the 2 cell types have different survival requirements. More focused experiments with conditional knockout models will be necessary to clarify how the regulation of splicing and transcription interact to cause such a cell type-specific phenotype.

### 4.3 SFSWAP

Another hearing-related splicing regulator was discovered in a lentiviral mutagenesis screen. The study identified a mouse strain that exhibited circling behavior because of a mutation in an intron of the *Sfswap* gene (Moayedi et al., 2014). First identified in *Drosophila*, SFSWAP (SFRS8) is an RBP that regulates the splicing of several genes, including *Sfswap* itself (Chou et al., 1987; Zachar et al., 1987; Sarkissian et al., 1996). Mutant mice exhibited hearing and balance deficits, possessed shorter cochleae and smaller vestibules than wildtype, and had fewer third row OHCs and SCs, as well as some ectopic IHCs (Moayedi et al., 2014). The authors noted that this phenotype was similar to that observed in mice heterozygous for *Jag1*, a Notch ligand crucial for cochlear sensory development (Kiernan et al., 2001). The severe functional and morphological defects seen in *Sfswap* ^−/−^;*Jag1* ^+/−^ mice were argued to be evidence of a synergistic relationship between the two genes (Moayedi et al., 2014). Although a subsequent RT-PCR experiment revealed the downregulation of some Notch pathway genes, the expression of *Jag1* itself was unaffected. Neither were any splicing defects observed in the affected genes (Moayedi et al., 2014). Since then, only one further study of the *Sfswap* mutant has been published, and it concerned the biophysical properties of the mutant cochleae (Gao et al., 2014). It remains unclear whether splicing dysregulation is even occurring at all. Transcriptome-wide studies will probably be necessary to confirm this, as well as to identify the underlying mechanism.

### 4.4 RBM24

RBM24 is an RBP that was first implicated in muscle development, but RBM24 expression was also found *via* immunostaining in HCs of both the cochlea and vestibule (Grifone et al., 2018). *Rbm24* has been identified as a target of ATOH1 and GFI1, key transcription factors required for HC differentiation, through DNA-protein interaction assays and *in vivo* knockout studies (Cai et al., 2015; Jen et al., 2022). Whole-body deletion of *rbm24a* in zebrafish embryos disrupted HC morphogenesis and led to hearing and balance deficits (Zhang et al., 2020a; Cheng et al., 2020). Deletion of *Rbm24* in 2-month-old mice also resulted in auditory and vestibular dysfunction (Zheng et al., 2021). Interestingly, although *Rbm24* was expressed in both types of HCs, its mutation selectively affected OHCs (Zheng et al., 2021). This cell type-specific phenotype was replicated in a mouse study that deleted *Rbm24* in all cells that express *Atoh1*, including cochlear HCs. Although OHC numbers were normal at P0, they were greatly reduced by P19 (Wang et al., 2021). Thus, this RBP seems to be important for postnatal survival of OHCs in the cochlea.

Since RBM24 immunofluorescence was mainly observed in the cytoplasm in head sensory epithelia, it was initially suggested that the protein plays a role in mRNA stabilization (Grifone et al., 2018). Consistent with this prediction, RNA-sequencing revealed no significant splicing changes in the otic vesicles of *rbm24* mutant zebrafish. This was despite many changes in gene expression, including those in the expression of several orthologues of human deafness genes (Zhang et al., 2020a). Nevertheless, it was previously demonstrated *in vitro* that RBM24 regulated AS of *CDH23* exon 68 (Li et al., 2020a). RT-PCR revealed that inclusion of *CDH23* exon 68 was indeed reduced in *rbm24* mutant zebrafish (Zhang et al., 2020a). In addition, several RBM24-dependent alternative exons were identified in mice, including *Cdh23* exon 68 and *Kcnq4* exon 9 (Zheng et al., 2021). Some of these exons seem to be cochlea-specific, and their corresponding genes include Usher genes (*Myo7a*, *Ush1c, Pcdh15*), hair bundle link genes (*Ptprq*), and ion channel genes (*Cacna1d*, *Atp2b2*) (Zheng et al., 2021).

In summary, it is far from clear whether the phenotypes of RBM24-deficient cochleae can be attributed to splicing dysregulation. Although the inclusion of *Cdh23* exon 68 does seem to be genuinely regulated by RBM24, there is ample residual expression of the exon 68-containing isoform in mutant cochleae to cast doubt on its implication in hearing pathogenesis (Zhang et al., 2020a; Zheng et al., 2021). The other AS events mentioned in the last paragraph should also be validated. Because RBM24 participates in a variety of post-transcriptional regulatory mechanisms in other tissues (Grifone et al., 2020), splicing-independent mechanisms must also be considered. For example, regulation of *p53* mRNA translation by RBM24 is required to maintain appropriate levels of programmed cell death during heart development (Zhang et al., 2018). Comparable mechanisms are likely at play in the postnatal OHCs. This example illustrates the difficulty of inferring the action of an RBP from the phenotypes of its loss-of-function mutant models.

## 5 Aberrant splicing as a cause of hearing loss in humans

When splicing regulatory elements are disrupted, the ensuing aberrant splicing can cause disease. Mutations affecting splice site sequences or *cis*-regulatory elements are the most common splice-altering mutations, and indeed a significant proportion of the mutations associated with hearing loss belong to this category (Abu Rayyan et al., 2020). In contrast, mutations affecting *trans*-regulatory elements appear more rarely because they simultaneously affect the splicing of many genes.

An interesting example was described in a recent study of a pedigree of recessively inherited non-syndromic hearing impairment. A synonymous variant was identified in *TMC1*, which had not been labeled pathogenic according to previous classifications because it was neither predicted to affect existing splice site sequences nor to activate new ones. Further *in silico* analysis, however, suggested the mutation may disrupt exonic *cis*-regulatory elements. On the basis of *in vitro* experiments confirming the predicted splicing alteration, the authors proposed that the variant be reclassified as pathogenic (Vaché et al., 2022). This example illustrates the importance of taking splicing into consideration when evaluating variant pathogenicity.

It is obvious that aberrant splicing of essential cochlear genes would impair hearing, but such instances do not necessarily demonstrate the relevance of AS in a physiological context. If the splicing event in question occurs only in pathological conditions, all we can learn from the phenotype is that the gene is actually required for hearing. Therefore, these events will not be discussed further in this review. Nevertheless, some cases of genetic hearing loss do provide insight into the role of AS in cochlear physiology. For example, some hearing disorders are caused by mutations in alternative exons. Moreover, their clinical manifestations sometimes depend on the isoforms affected by the mutation. Even if the mutations are not splice-altering in nature, they allow us to infer the role of each splice isoform by examining the phenotype resulting from its specific disruption. Several such examples are summarized in Table 1.

**TABLE 1:** Mutations associated with hearing loss that affect specific splice variants.

Gene	Location of mutation	Molecular consequence	Associated phenotype	References
*USH1C*	Exons 3 & 5, intron 5, intron 5/exon 6 junction, *etc*.	Constitutive exons are affected	USH1C; congenital severe-to-profound hearing loss along with retinitis pigmentosa	Verpy et al. (2000); Ouyang et al. (2003); Ebermann et al. (2007a)
	Exons B, D	Additional domains translated only in class b isoforms are affected	DFNB18; prelingual severe-to-profound sensorineural hearing loss	Ouyang et al. (2002)
	Exon 15	Class a and c isoforms are affected	Retinitis pigmentosa, together with mild late onset hearing loss	Khateb et al. (2012)
*PCDH15*	Exons 6, 17, 32, *etc*.	All cytoplasmic isoform classes are affected	USH1F; congenital severe-to-profound hearing loss along with retinitis pigmentosa	Ahmed et al. (2008)
	Exon 38	Only CD2 isoforms are affected	Non-syndromic congenital, profound sensorineural hearing loss	Pepermans et al. (2014)
*USH2A*	Exons 22–72	Only the long, transmembrane isoforms are affected	USH2A; congenital moderate-to-severe hearing loss along with retinitis pigmentosa, usually without vestibular dysfunction	Aller et al. (2006); Dai et al. (2008); McGee et al. (2010); Xu et al. (2011)
*MYO15A*	Exon 2 (all mutations are truncating mutations)	Isoforms including the large N-terminal domain are truncated	DFNB3; severe-to-profound hearing loss. The striking absence of pathogenic mis-sense mutations in exon 2 implies a milder, clinically overlooked phenotype	Nal et al. (2007); Rehman et al. (2016)
*TRIOBP*	Exons 7 & 9 (compound)	One copy of TRIOBP-4 is functional, while TRIOBP-5 and 6 isoforms are completely deleted	DFNB28, but with a milder or later-onset phenotype compared to the usual congenital, profound hearing loss	Pollak et al. (2017); Wesdorp et al. (2017)
*ATP2B2*	Exon 8	Isoform w is affected while x and z remain intact	DFNA82; childhood-onset, rapidly progressive sensorineural hearing loss	Smits et al. (2019)
*OTOF*	Exon 48	Highly conserved C-terminal domain of the major cochlear isoform is disrupted	AUNB1; non-syndromic autosomal recessive auditory neuropathy	Yasunaga et al. (2000); Rodríguez-Ballesteros et al. (2003); Varga et al. (2003); Choi et al. (2009)

In this section, we will first look at some splice-altering mutations that cause hearing loss. As we explained, the existence of non-syndromic deafness caused by mutations in *cis*-regulatory sequences of essential hearing genes is unsurprising. We will focus on cases that could be expected to produce a global phenotype but that present with the specific symptom of hearing loss. These kinds of examples are opportunities to clarify the unique properties of the cochlea. After exploring rare cases of hearing loss arising from the dysregulation of *trans-*regulatory elements or of the core splicing machinery, we discuss intriguing reports in which efforts to clarify the function of deafness-associated genes have led to unexpected discoveries suggesting their involvement in splicing. Finally, we explore how the splicing might be engineered to tackle various hearing loss disorders.

### 5.1 Alterations of *cis*-regulatory elements

#### 5.1.1 DFNA5: A gain-of-function splice alteration

DFNA5 is a form of autosomal dominant non-syndromic sensorineural hearing loss that arises *via* a gain-of-function mechanism related to the Gasdermin E gene (*GSDME*). Interestingly, *Gsdme* knockout mice exhibit normal hearing (Van Laer et al., 2005), as do humans with whole gene deletion and truncation mutations in exon 5 (Dunø et al., 2004; Van Laer et al., 2007). Every pathogenic *GSDME* mutation identified thus far has been found to cause exon 8 skipping. These variants include disruptions in the consensus splice acceptor site (Bischoff et al., 2004; Chai et al., 2014; Wang et al., 2018; Chen et al., 2020; Yuan et al., 2020; Mansard et al., 2022), the splice donor site (Cheng et al., 2007; Li-Yang et al., 2015), the intronic splicing regulatory elements (Van Laer et al., 1998; Yu et al., 2003; Park et al., 2010; Nishio et al., 2014; Nadol et al., 2015; Booth et al., 2018b; Booth et al., 2020), or exonic splicing regulatory elements (Booth et al., 2018b).

The skipping of exon 8 leads to a frameshift and C-terminal truncation, and expression of this truncated GSDME caused cell death (Van Laer et al., 2004; Op de Beeck et al., 2011; Van Rossom et al., 2012). Wildtype GSDME protein was later found to comprise a cytotoxic N-terminal domain, a connecting hinge region, and a self-inhibitory C-terminal domain. Cleavage of the hinge releases the cytotoxic N-terminal domain, triggering inflammatory cell death (Rogers et al., 2017; Zhang et al., 2020b). Together, these results suggest constitutive cytotoxic activity of mutant GSDME may underlie DFNA5, with enhanced cell death eventually manifesting as sensorineural hearing loss. Although there is no direct evidence, the progressive nature of the hearing impairment in DFNA5 is consistent with this hypothesis (Op de Beeck et al., 2012). Also, histopathologic examination of temporal bone specimens from one DFNA5 patient revealed loss of cochlear HCs, as well as degeneration of spiral ganglion neurons and various regions of the inner ear epithelium (Nadol et al., 2015).

It remains unclear, however, why activation of GSDME promotes enough cell death in the cochlea to produce a discernible phenotype but not in other organs. It is possible that GSDME is simply expressed at low levels in other organs. Consistent with this hypothesis, one study found intense expression of *GSDME* mRNA in the placenta and cochlea and minimal expression in the heart, brain, and kidney (Van Laer et al., 1998). It is also possible that the cochlea is particularly susceptible to degeneration. Consistent with this hypothesis is the fact that cochlear HCs are notoriously resistant towards attempts to trigger their regeneration (Burns and Corwin, 2013).

#### 5.1.2 DFNA27: Tissue-specific requirements for AS

The next example concerns a gene already discussed above—*REST*. In a forward genetic screen, a pathogenic locus for an autosomal dominant form of non-syndromic hearing loss was mapped to the *REST*-containing region of chromosome 4 and designated *DFNA27* (Peters et al., 2008). But no pathogenic variants were identified in the exons of *REST*, nor did heterozygous knockout of *REST* result in hearing loss (Gao et al., 2011). Later, a variant in a conserved intronic region of *REST* was found in a pedigree analysis to co-segregate with hearing loss. The variant promoted the usage of an aberrant splice site at *REST* exon 4a/b. Normally, SRRM4 would promote inclusion of exon 4a/b to produce truncated, inactive proteins, but use of the aberrant splice site in the presence of SRRM4 generates a mutant form of exon 4b. This mutant exon 4b does not introduce any stop codons and enables translation of the full-length product, albeit with some amino acid sequence differences (Nakano et al., 2018).

Downregulation of REST is required, not only in HCs, but also in neurons (Mandel et al., 2011; Baldelli and Meldolesi, 2015; Lu et al., 2018). How is it, then, that a REST gain-of-function mutation leads to a cochlea-specific phenotype? As mentioned previously, OHCs transiently repress *REST* transcription independently of SRRM4 and are thought to be spared from degeneration for that reason (Nakano et al., 2020). Similarly, differentiating neurons employ a variety of strategies other than AS to ensure robust inactivation of REST, including transcriptional silencing, ubiquitination, and phosphorylation (Nesti et al., 2014). Thus, in this case, the tissue-specific nature of the phenotype seems to arise from differential requirements for AS.

#### 5.1.3 DFNB39: Tissue-specific splicing at play?

DFNB39, an autosomal recessive form of non-syndromic sensorineural hearing loss (Wajid et al., 2003), is caused by mutations in the hepatocyte growth factor gene (*HGF*). Three potentially pathogenic variants have been reported. One is a synonymous substitution that led *in vitro* to greatly reduced use of exon 5a relative to exon 5b. The other two variants are deletions in a conserved intronic region transcribed as the 3′-UTR of a short splice variant of *HGF* (Schultz et al., 2009). Mice homozygous for one of the deletions faithfully reproduced the early-onset non-syndromic hearing loss phenotype of DFNB39, which was attributed to defective incorporation of melanocytes into the stria vascularis due to disruption of HGF-MET signaling (Shibata et al., 2016; Morell et al., 2020).

Inquiries into this tissue-specific phenotype led to some intriguing findings. Although HGF is normally expressed at high levels in organs other than the cochlea (e.g., lung, kidney), these organs were normal in homozygous knock-in mice. Comparisons between these organs revealed that the intronic mutation reduced *Hgf* mRNA expression in the cochlea, but not in the lung or kidney. When transcripts including exons 6a and 6b (the mouse equivalents of human exons 5a and 5b) were quantified separately, the use of exon 6a was reduced in the cochleae of mutant mice but not in other organs (Morell et al., 2020).

It is tempting to assume that this reflects tissue-specific regulation of splicing. The intronic deletion may require cochlea-specific *trans*-regulatory elements to affect splicing decisions. Considering that all three pathogenic variants reduce the inclusion of exon 5a, functional differences among the various isoforms may also contribute to tissue specificity. We must also consider mutations in the *MET* gene, which encodes an HGF receptor, mutations of which can lead to non-syndromic hearing loss (DFNB97) (Mujtaba et al., 2015; Bousfiha et al., 2019). As HGF-MET signaling is implicated in a myriad of biological processes (Baldanzi and Graziani, 2015), it is surprising that mutations of both *HGF* and *MET* can show such tissue-specific effects on the cochlea. Perhaps cochlear development requires specific isoforms of a component of the HGF-MET signaling pathway. Regardless of its mechanism, this is an interesting instance of tissue-specific AS co-occurring with, if not causing, a cochlea-specific phenotype.

### 5.2 Alterations of *trans*-regulatory elements

As mentioned above, mutations in *ESRP1*, a splicing factor and regulator of cochlear non-sensory development, have been associated with hearing loss. Not only was the splicing of ESRP1-dependent cassette exons altered in patient-derived induced pluripotent stem cells carrying *ESRP1* mutations, but the splicing defects were restored when the *ESRP1* mutations were corrected. In fact, this very finding was the motivation for an investigation into the role of this splicing factor in cochlear development (Rohacek et al., 2017). To this day, this remains the only instance where mutations in a known splicing regulator gene have been implicated in human hearing loss.

### 5.3 Dysregulation of the core splicing machinery

Mutations affecting the core splicing machinery can also cause disease. Since core spliceosome components are, by definition, required constitutively in every cell, their disruption usually results in embryonic lethality. In some cases, however, their disruption can preferentially affect some cell types over others, the most prominent example being neural crest cells (Beauchamp et al., 2020). This implies that certain cells are particularly vulnerable to splicing dysregulation (Olthof et al., 2022).

Hearing loss is a component of several developmental disorders caused by mutations in core splicing machinery genes (Ritter and Martin, 2019). Does this imply that cochlear cells are particularly vulnerable to aberrant splicing? Rather than arising from defective splicing in the cochlea, these developmental forms of hearing loss are more likely caused by defective neural crest cell migration into the stria vascularis where they normally form melanocytes (Ritter and Martin, 2019). In this regard, an interesting observation was made in a study of a zebrafish model of Nager syndrome. Maharana and Saint-Jeannet, (2021), noting that Nager and Rodriguez syndrome patients typically exhibit hearing loss, hypothesized that the splicing factor Sf3b4 might be necessary for cochlear development. *Sf3b4* depletion in zebrafish led to reduced expression of genes related to development of the otic placode—the embryonic structure that develops into the otocyst. After also observing smaller otocysts in *Sf3b4* mutant zebrafish, the authors concluded that Sf3b4 is important in otic placode in addition to neural crest development (Maharana and Saint-Jeannet, 2021). It remains unclear whether this is a first hint at an inner ear-specific requirement for correct splicing regulation or merely a non-specific phenomenon that should be expected to accompany housekeeping gene deletion.

### 5.4 Splice-altering activity of hearing-related proteins

#### 5.4.1 SANS

Recently, it has been suggested that splicing dysregulation may play a role in the pathogenesis of Usher syndrome (Yildirim et al., 2021). While the hearing loss of Usher syndrome arises from dysregulation of stereocilia development, the pathological mechanism of its associated retinitis pigmentosa remains rather unclear. Yildirim et al. (2021) found *via* yeast two-hybrid screening that the Usher protein SANS (USH1G) may interact with several components of the spliceosome (Yildirim et al., 2021). They then found that knockdown of *SANS in vitro* resulted in slower splicing kinetics and altered inclusion of several cassette exons. Most interestingly, two of the exons they studied belonged to the *MYO7A* and *USH1C* genes. The authors then proposed a model in which SANS promotes the transportation of spliceosome components to nuclear speckles, where they are stored temporarily before being recruited to sites of active spliceosome assembly (Yildirim et al., 2021).

*In vivo* confirmation of these results would constitute compelling evidence for a role for SANS in splicing regulation, but it remains unclear whether such a mechanism is necessary in accounting for the auditory phenotype of Usher syndrome. SANS interacts with other Usher proteins to form the scaffolds that support the stereociliary tip links (Caberlotto et al., 2011; Grati and Kachar, 2011). In mouse models, SANS depletion leads to abnormal stereocilia development in HCs (Kitamura et al., 1992). Although defective stereocilia morphogenesis seems to be the primary pathology in the Usher syndrome cochlea, it is possible that aberrant splicing of Usher genes due to SANS depletion also contributes to hair bundle malformation. Alternatively, splicing dysregulation could represent an entirely different mechanism underlying Usher syndrome pathogenesis. Another recent report of an interaction between splicing-related proteins and the Usher protein VLGR1 lends more credibility to the latter suggestions (Knapp et al., 2022). It will be interesting to see how these findings generalize to the retinae and cochleae of live animal models.

#### 5.4.2 ILDR1

Efforts to uncover the pathogenesis of DFNB42, a non-syndromic hearing loss disorder caused by mutations in the *ILDR1* gene, also led to some unexpected discoveries (Borck et al., 2011; Liu et al., 2017). *Ildr1* knockout mice are profoundly deaf due to loss of cochlear HCs (Higashi et al., 2015; Morozko et al., 2015; Sang et al., 2015), but what causes the degeneration? Initial investigations that focused on the role of ILDR1 in cochlear tricellular junctions (tTJs) were rather inconclusive (Higashi et al., 2015; Morozko et al., 2015). Using a yeast two-hybrid approach to search for alternative ILDR1 functions, one group discovered that ILDR1 binds the splicing factors TRA2A, TRA2B, and SRSF1, translocating into the nucleus when these factors are present (Liu et al., 2017). They found ILDR1 affected the AS of *Tubd1*, *Iqcb1*, and *Pcdh19* transcripts *in vitro*, but they were unable to discern any differences in the splicing patterns of wildtype and *Ildr1* knockout mouse cochleae. Since ILDR2 interacted with the same splicing factors as ILDR1 and similarly affected AS in cultured cells, compensatory upregulation of ILDR2 could account for the discordance. Selective and simultaneous knockdown of *ILDR1* and *ILDR2* in cells indicated that the effects of the two proteins on splicing were additive (Liu et al., 2017).

Is splicing dysregulation an additional pathway that contributes to epithelial barrier disruption? Or does it affect hearing through an altogether different mechanism? Since the proposed target genes of ILDR1 have not been implicated in hearing, more unbiased analyses may be necessary to identify relevant pathways. The hypothesis proposed by the authors will have to be tested by generating mice doubly deficient for ILDR1 and ILDR2.

### 5.5 Antisense oligonucleotides for treating hearing loss

An emerging therapeutic strategy against splicing dysregulation caused by mutations in *cis*-regulatory elements is the delivery of antisense oligonucleotides (ASOs). Splice-switching ASOs are short oligonucleotides designed to bind specific RNA sequences and alter splicing, preventing the production of pathogenic transcripts. Splice-switching ASOs are actively being developed with the aim of treating hearing loss arising from *cis*-acting mutations (Hastings and Jones, 2019). We will discuss some prominent examples in the following paragraphs.

#### 5.5.1 Usher syndrome

A G>A mutation at position c.216 in *USH1C* exon 3 accounts for most cases of type 1 Usher syndrome in Acadian and related populations (Ouyang et al., 2003; Ebermann et al., 2007a). This mutation strengthens a latent 5′splice site, facilitating its preferential utilization over the constitutive splice site. As a result, a premature termination codon is introduced and truncated proteins are produced (Lentz et al., 2005). Mice homozygous for this mutation were deaf and displayed behavior suggestive of vestibular dysfunction. When a splice-switching ASO complementary to the aberrant splice site sequence was injected intraperitoneally at P5, the mis-splicing of *Ush1c* was partially corrected and hearing was restored. This rescue was complete at lower frequencies and partial at higher frequencies, persisting for at least 6 months. Vestibular dysfunction was also ameliorated (Lentz et al., 2013; Vijayakumar et al., 2017; Donaldson et al., 2018). This was the first demonstration in an animal model of the potential of ASOs for treating inner ear dysfunction caused by a mutation identified in human patients. Several groups are working continuously to optimize the timing (Vijayakumar et al., 2017; Donaldson et al., 2018; Ponnath et al., 2018) and route (Depreux et al., 2016; Lentz et al., 2020; Wang et al., 2020) of ASO administration in hope of extending their application to human patients.

Analogous strategies have been pursued for other types of Usher syndrome (Liquori et al., 2016; Slijkerman et al., 2016; Slijkerman et al., 2018; Panagiotopoulos et al., 2020; Dulla et al., 2021), although none have focused on the inner ear phenotype. One specific ASO construct is worth discussing because it illustrates the important principle that splice-switching ASOs can be effective even when the mutation in question is not splice-altering in nature and because it is closest to being used in the clinic as we write this article. Ultevursen, which was previously dubbed QR-421a, was designed to induce the skipping of *USH2A* exon 13 (Dulla et al., 2021). Truncating mutations in exon 13, such as c.2299delG, account for a large proportion of type 2A Usher syndrome cases (Yan et al., 2009). Skipping this exon does not cause a frameshift, and so it leads to the translation of partially functional proteins. Positive results were reported in a phase 1/2 clinical trial evaluating the safety and tolerability of ultevursen in retinitis pigmentosa patients (Audo et al., 2022), and a phase 2/3 clinical trial is currently under way (https://clinicaltrials.gov/ct2/show/NCT05158296).

#### 5.5.2 DFNB4/pendred syndrome

Mutations in the *SLC26A4* gene can cause both non-syndromic (e.g., DFNB4) and syndromic (e.g., Pendred syndrome, PS) forms of deafness (Li et al., 1998). The c.919–2A>G mutation of the *SLC26A4* gene is one of the most frequent mutations causing deafness in East Asian populations (Park et al., 2005; Du et al., 2013). This mutation, which is located at the 3′splice site of intron 7, promotes exon 8 skipping, which, in turn, introduces a premature termination codon (Yang et al., 2005). One study, using a multi-step screening process, identified an ASO that could correct a splicing defect in patient-derived blood cells and in mouse models (Feng et al., 2022). The ASO was predicted to prevent the binding of splicing silencers to a regulatory element located within intron 8 (Feng et al., 2022). Another study evaluated the feasibility of using small molecules to correct deafness-causing splicing defects (Lee et al., 2019). Some *SLC26A4* mutations occur in 5′splice sites where they are predicted to hinder recognition by the U1 snRNA, a component of the core splicing machinery. Synthetic U1 snRNAs were created with sequences perfectly complementary to those of the mutated splice sites. In many cases, these modified U1 snRNAs prevented mis-splicing, but some mutations near exon 8 were resistant to this type of treatment. In the presence of these resistant mutations, a nearby cryptic splice site was being used instead. To reduce competition for U1 snRNA binding, ASOs designed to mask the cryptic splice site were delivered together with the modified U1 snRNAs, and this restored normal splicing of exon 8 (Lee et al., 2019). These results show that different strategies for modulating splicing can be used together to produce a synergistic effect.

## 6 Conclusion

In this review, we covered many clues that point to the importance of AS in the cochlea. Both unbiased studies and targeted approaches will be necessary to proceed further. Unbiased high-throughput studies will, first of all, help to quantify the extent to which AS contributes to the unique transcriptomic landscape of the cochlea, which still has not been adequately assessed. The task of identifying AS events that occur differentially either along the tonotopic axis or among the various cochlear cell types will also benefit from such unbiased studies. In addition, uncovering the network of splicing regulators that govern cochlear development will require further unbiased analysis of gene expression, transcript usage, and RBP motif distribution. Meanwhile, as more AS events are identified, targeted investigations will be required to identify their full physiological significance. Examples of AS events that demand interpretation include the usage of *CDH23* exon 68 and the tonotopic splicing of *KCNMA1* and *KCNQ4*. Clarifying the precise ways RBPs such as SRRM4, SFSWAP, and RBM24 affect cochlear function is yet another task where much work remains.

Transcriptome studies of the cochlea have typically been limited by its small size and considerable heterogeneity. In bulk RNA-sequencing, the diverse cell types within each sample are all lumped together, while the high levels of dropout in single cell sequencing data make it difficult to computationally quantify isoform abundance with any accuracy (Buen Abad Najar et al., 2020; Westoby et al., 2020). Yet as in all fields, technological advances promise new opportunities. Methods such as fluorescence-activated cell sorting allow bulk RNA-sequencing to be applied at higher resolution, rendering it more suitable for inspecting heterogeneous tissues like the cochlea (Liu et al., 2018; Hertzano et al., 2021). On the other hand, single cell long-read RNA sequencing pipelines are continuously being refined, and the algorithms used to analyze their output are rapidly improving (Olivieri et al., 2022). As these tools become more powerful and effective, it will soon be possible to assess cochlear AS at sufficiently high resolution to draw more clear conclusions.

New venues of research are constantly opening up. For example, single cell-RNA sequencing has revealed unexpected cell type heterogeneity within the cochlear epithelium (Kelley, 2022) or the auditory ganglion (Petitpré et al., 2018; Shrestha et al., 2018; Sun et al., 2018). It would be worthwhile to investigate the contribution of AS to this newly discovered transcriptomic diversity. Also, splicing outcomes are governed not only by the mRNA sequence and the presence of *trans*-acting regulators, but also by chromatin structure and transcription kinetics (Kornblihtt et al., 2013). The interaction between splicing, transcription kinetics, and epigenetics could be investigated using recent techniques that allow simultaneous analysis of gene expression and chromatin accessibility at single-cell resolution. Finally, there are mechanisms other than AS that merit investigation as sources of molecular diversity. Many isoforms of hearing-related proteins are produced *via* alternative transcription/translation site usage, and they show different expression patterns (Sekerkova et al., 2006; Li et al., 2020b), subcellular localization patterns (Ebrahim et al., 2016), and associations with clinical phenotypes (Mburu et al., 2003; Tlili et al., 2005; Ebermann et al., 2007b; Audo et al., 2011; Besnard et al., 2012; Mathur et al., 2015). Isoform sequencing technologies could be employed to examine how the various post-transcriptional regulatory mechanisms contribute to molecular diversity in the cochlea. In summary, clarifying the role of AS in the cochlea is a viable goal that offers the enticing prospect of understanding the mechanisms that underlie the formation and function of transcriptomic diversity in the cochlea.
