# Supplementary material for: Alternative splicing in shaping the molecular landscape of the cochlea
Source: Front Cell Dev Biol. 2023 Mar 2;11:1143428. doi: 10.3389/fcell.2023.1143428 (PMC10018040; doi:10.3389/fcell.2023.1143428)
Supplement: Supplementary file 1 [file Table1.docx]

Supplementary Material

Supplementary Table S1. Known splice variants of genes related to cochlear structure or function. In the leftmost block, a brief overview of biological and clinical knowledge regarding each gene and the encoded protein is provided. The next two columns list and define the relevant splice variants, and explain the consequences of the AS events in question either at the transcript level or at the protein level. If there is any evidence that the splice variants exhibit different expression patterns or molecular properties (e. g., interaction with other proteins, subcellular localization, or electrophysiological parameters), they are presented in the next two columns. Finally, results from experiments using isoform-specific knockout animal models or clinical observations of patients with isoform-specific mutations are documented in the rightmost column.

# Abbreviations

AS: Alternative splicing

AUNB: Autosomal recessive auditory neuropathy

CDI: Calcium-dependent inactivation

DFNA: Autosomal dominant nonsyndromic hearing loss

DFNB: Autosomal recessive nonsyndromic hearing loss

HC: Hair cell

IHC: Inner hair cell

LTLD: Lower tip link density

MET: Mechanoelectric transduction

OHC: Outer hair cell

PTC: Premature termination codon

UTLD: Upper tip link density

UTR: Untranslated region

| **Usher and related proteins** | | | | | | |
| --- | --- | --- | --- | --- | --- | --- |
| **Gene** | **Encoded protein** | **Splice variants** | **Structural consequences** | **Differential expression** | **Molecular properties** | **Relevance *in vivo*** |
| *USH1C* | Harmonin | Splice variants are categorized into three classes, a, b, and c. Class a includes the canonical transcript. | Compared to the canonical isoform, harmonin-b contains additional functional domains encoded by alternative exons. Class c isoforms lack the third PDZ domain normally present in the canonical isoform, because their transcripts contain PTCs. | Harmonin-a is expressed rather broadly; it is present both in the inner ear and in the retina. On the other hand, certain transcripts belonging to class b are expressed specifically in the inner ear and some other tissues, but not in the eye. | Harmonin-a and harmonin-b show distinct subcellular localizations and interaction properties. Consult the text for details. | Specific deletion of harmonin-b in mice affected hair bundle morphology and MET current kinetics in OHCs (Michalski et al., 2009). Usher syndrome phenotypes differ according to the affected isoform (Ouyang et al., 2002; Khateb et al., 2012). |
| **Description of protein** | |  |  |  |  |  |
| A scaffold protein that is the hub of the Usher protein interaction network | |  |  |  |  |  |
| **Associated disorders and models** | |  |  |  |  |  |
| Usher syndrome type 1C DFNB18 *Deaf circler* (*dfcr*) mouse | |  |  |  |  |  |
| **Gene** | **Encoded protein** | Three types of transcripts that differ in their number of extracellular cadherin repeats arise via utilization of alternative promoters. All three types can be subdivided according to the inclusion of the alternative exon 68. | Although initially supposed as disrupting an internal PDZ domain-binding motif, the region encoded by exon 68 turned out to be a domain that can bind the N-terminal domain of harmonin (Wu et al., 2012). Consult the text for details. | Inclusion of the alternative exon 68 has repeatedly been shown to be inner ear-specific. | Inclusion of exon 68 is predicted to affect the conformation and interaction properties of the protein. Consult the text for details. |  |
| *CDH23* (*USH1D*) | CDH23 |  |  |  |  |  |
| **Description of protein** | |  |  |  |  |  |
| Component of the stereociliary tip link that anchors it to the UTLD | |  |  |  |  |  |
| **Associated disorders and models** | |  |  |  |  |  |
| Usher syndrome type 1D DFNB12 *Waltzer* (*v*), *salsa*, *modifier-of-deafwaddler* (*mdfw*), and *age-related hearing loss* (*Ahl*) mice | |  |  |  |  |  |
| **Gene** | **Encoded protein** | At least 24 splice variants are broadly classified into 3 categories, CD1-3, according to the inclusion of either exon 35, 38, or 39, respectively. Also, a large variety of N-terminal splice variants exist. | CD1-3 isoforms differ in their cytoplasmic domains, which all contain a PDZ domain-binding motif. The number of extracellular cadherin repeats varies widely as the result of alternative N-terminal splicing. | In humans, CD3 isoforms are broadly expressed whereas CD1 and CD2 are expressed in the cochlea, retina, and a few other tissues (Ahmed et al., 2008). Also, the isoforms show different temporal expression patterns during cochlear development (Ahmed et al., 2006; Michel et al., 2020) . | Isoforms differ in their interactions with TMIE (Zhao et al., 2014) as well as in intracellular trafficking tendencies (Ballesteros et al., 2022). Extracellular domain variants exhibit different binding affinities towards CDH23, with that of the canonical isoform being the strongest (Narui and Sotomayor, 2018). | CD1, 2, and 3 isoforms compensate for each other during early stereocilia development, but only CD2 isoforms are specifically required for hearing in mature HCs (Webb et al., 2011; Pepermans et al., 2014). Mutations that selectively affect the CD2 isoforms have been found in two families with nonsyndromic hearing loss (Pepermans et al., 2014). |
| *PCDH15* (*USH1F*) | PCDH15 |  |  |  |  |  |
| **Description of protein** | |  |  |  |  |  |
| Component of the stereociliary tip link that anchors it to the LTLD | |  |  |  |  |  |
| **Associated disorders and models** | |  |  |  |  |  |
| Usher syndrome type 1F DFNB23 *Ames waltzer* (*av*) mouse | |  |  |  |  |  |
| **Gene** | **Encoded protein** | **Splice variants** | **Structural consequences** | **Differential expression** | **Molecular properties** | **Relevance *in vivo*** |
| *USH2A* | Usherin | The gene was first described as consisting of 21 exons, but 51 novel exons at the 3' end were later identified (van Wijk et al., 2004). The original isoform is termed class a, and long isoforms containing later-identified exons are categorized as class b. | The short variant is predicted to encode a secreted protein (Bhattacharya et al., 2002), whereas long isoforms are transmembrane proteins with additional functional domains *e. g.*, a PDZ-binding motif located at its cytoplasmic domain (van Wijk et al., 2004). |  | The long isoform interacts directly via its PDZ-binding motif with whirlin and harmonin (Adato et al., 2005). | Mutations located in the additional exons have been found to be associated with Usher syndrome, thereby demonstrating the requirement for at least one long isoform (Aller et al., 2006; Dai et al., 2008; McGee et al., 2010; Xu et al., 2011). |
| **Description of protein** | |  |  |  |  |  |
| Component of the ankle link complex | |  |  |  |  |  |
| **Associated disorders and models** | |  |  |  |  |  |
| Usher syndrome type 2A | |  |  |  |  |  |
| **Gene** | **Encoded protein** | Short and long splice variants are defined according to whether the last 3 exons in the C-terminal side, exons 14-16, are included or not (Zou et al., 2014). | Compared to short isoforms that mostly contain two PDZ domains, long transcripts encode full-length proteins that additionally possess a proline-rich domain and one more PDZ domain (Zou et al., 2014). | In mice, long transcripts could be detected in the cochlea but not the retina, although this pattern could not be observed at the protein-level (Zou et al., 2014). | Only the long isoform localizes to ankle links and is involved in trafficking of other ankle link components (Du et al., 2020). | Selective depletion of the long isoform disrupted hair bundle morphology as well as MET currents, and caused hearing loss (Du et al., 2020). |
| *PDZD7* | PDZD7 |  |  |  |  |  |
| **Description of protein** | |  |  |  |  |  |
| Component of the ankle link complex | |  |  |  |  |  |
| **Associated disorders and models** | |  |  |  |  |  |
| DFNB57 Usher syndrome type 2 (as a modifier) | |  |  |  |  |  |
| **Gene** | **Encoded protein** | At least 3 splice variants have been reported (Västinsalo et al., 2011). |  |  |  |  |
| *CLRN* (*USH3A*) | Clarin-1 |  |  |  |  |  |
| **Description of protein** | |  |  |  |  |  |
| Required for both stereocilia and ribbon synapse functions | |  |  |  |  |  |
| **Associated disorders and models** | |  |  |  |  |  |
| Usher syndrome type 3A | |  |  |  |  |  |
| **Actin-related proteins** | | | | | | |
| **Gene** | **Encoded protein** | **Splice variants** | **Structural consequences** | **Differential expression** | **Molecular properties** | **Relevance *in vivo*** |
| *MYO15A* | Myosin XVa | Two classes of isoforms exist depending on the inclusion of exon 2. | Exon 2 encodes a large N-terminal domain. | The short isoform is predominant in early postnatal stages, but around P7~14 the major isoform switches to the long one (Fang et al., 2015). | Short isoforms are mainly trafficked to the tips of tallest stereocilia while the long ones are found in the remaining rows (Fang et al., 2015). | Mice without long isoforms show normal stereociliary recruitment of EPS8 and whirlin. The long isoform plays a role in actin maintenance at later stages (Fang et al., 2015). |
| **Description of protein** | |  |  |  |  |  |
| Localizes to stereociliary tips and probably recruits EPS8 and whirlin therein | |  |  |  |  |  |
| **Associated disorders and models** | |  |  |  |  |  |
| DFNB3 *Shaker 2* (*sh2*) mouse | |  |  |  |  |  |
| **Gene** | **Encoded protein** | **Splice variants** | **Structural consequences** | **Differential expression** | **Molecular properties** | **Relevance *in vivo*** |
| *MYH9* | Class II non-muscle myosin heavy chain-A (NMHC-IIA) | Variants with cassette exons inserted near the regions encoding either the ATP-binding domain (loop 1) or the actin-binding domain (loop 2) have been identified (Li et al., 2007). | Insertions in the loop 1 coding region introduce PTCs (Li et al., 2007). | Loop 1 insertions were detected in various organs including the cochlea, whereas the variant with loop 2 insertion was detected only in the brain and in the cochlea (Li et al., 2007). |  |  |
| **Description of protein** | |  |  |  |  |  |
| Expressed in the stereocilia, cuticular plate, cytoplasm and along the plasma membrane of cochlear HCs | |  |  |  |  |  |
| **Associated disorders and models** | |  |  |  |  |  |
| DFNA17 MYH9-related disease | |  |  |  |  |  |
| **Gene** | **Encoded protein** | TRIOBP-4 and 5 are generated through AS, whereas other isoforms arise via the use of alternative transcription start sites. |  | While TRIOBP-1 is ubiquitous, expression of TRIOBP-4, 5, and 6 is rather restricted, mainly to the inner ear and the retina. Both TRIOBP-4 and 5 are found in stereociliary rootlets. |  | While deletion of both TRIOBP-4 and 5 in mice result in failure of stereocilia formation and profound deafness, specific deletion of the long TRIOBP-5 isoform causes late-onset disorganization of stereocilia and manifests as progressive or mild hearing loss (Kitajiri et al., 2010; Katsuno et al., 2019). |
| *TRIOBP* | TRIOBP-1, 4, 5, 6 |  |  |  |  |  |
| **Description of protein** | |  |  |  |  |  |
| Involved in actin stabilization in HCs as well as in Deiters' cells (a type of SC) | |  |  |  |  |  |
| **Associated disorders and models** | |  |  |  |  |  |
| DFNB28 | |  |  |  |  |  |
| **Gene** | **Encoded protein** | Long transcripts containing exon 7 correspond to the canonical protein, whereas certain short transcripts called XELPINs splice out the exon. | XELPINs lack XIN-repeat domains. | Short isoforms are predominant in the inner ear, but are absent from the heart (Francis et al., 2015; Scheffer et al., 2015). | Stereocilia specifically utilize short isoforms, while long isoforms mainly seem to localize to cuticular plates (Francis et al., 2015; Scheffer et al., 2015). |  |
| *XIRP2* | XIRP2 |  |  |  |  |  |
| **Description of protein** | |  |  |  |  |  |
| The conventional protein is expressed in skeletal and cardiac muscle, whose 28 XIN domains confer actin crosslinking activity | |  |  |  |  |  |
| **Associated disorders and models** | |  |  |  |  |  |
| *Xirp2* knockout mice show high frequency hearing impairment (Francis et al., 2015) | |  |  |  |  |  |

| **Components of the MET complex** | | | | | | |
| --- | --- | --- | --- | --- | --- | --- |
| **Gene** | **Encoded protein** | **Splice variants** | **Structural consequences** | **Differential expression** | **Molecular properties** | **Relevance *in vivo*** |
| *TMC1* | TMC1 | Two types of transcripts have been detected in the mouse cochlea, *Tmc1ex1* and *Tmc1ex2*, the latter of which includes the alternative exon 2 (Kawashima et al., 2011). | The translation start sites of the two transcripts are located within exon 1 and 2, respectively (Kawashima et al., 2011). A short upstream open reading frame and a weak kozak sequence renders translation from *Tmc1ex2* difficult (Yamaguchi et al., 2020). | *Tmc1ex2* is considerably rarer than *Tmc1ex1* in adult cochleae (Yamaguchi et al., 2020), but is expressed in comparable levels during the early postnatal period (Kawashima et al., 2011). |  |  |
| **Description of protein** | |  |  |  |  |  |
| Leading candidate for the pore-forming component of the MET complex | |  |  |  |  |  |
| **Associated disorders and models** | |  |  |  |  |  |
| Dominant mutations cause DFNA36 while recessive mutations cause DFNB7/11. Analogous mouse models exist, named *Beethoven* (*Bth*) and *deafness* (*dn*), respectively. | |  |  |  |  |  |
|  |  | Two additional AS events have been detected — exon 9 skipping and usage of an alternative 3' splice site of intron 14 (Zhou et al., 2021). | Regions disrupted by the two events correspond to the first putative extracellular loop and the sixth transmembrane domain, respectively (Zhou et al., 2021). | The events were of low frequency, but were more prevalent in OHCs than in IHCs. The skipped exon event seemed to be specific to the cochlea and testis (Zhou et al., 2021). |  |  |
| **Gene** | **Encoded protein** | An alternative 3' splice site usage event at exon 2 was fortuitiously detected in mice homozygous for the *hscy-2J* mutation, a recessive allele discovered in a mutagenesis screening process (Longo-Guess et al., 2007). | Part of the second extracellular loop, the fourth transmembrane domain, and most of the C-terminus is deleted (Zhou et al., 2021). | The event seemed to be specific to the cochlea and testis (Zhou et al., 2021). |  | Heterozygotes were normal, while homozygotes were profoundly deaf (Longo-Guess et al., 2007). This was taken as evidence that the variant was functionally null, but it has been argued that since the wildtype allele produces the variant albeit at low levels, some dominant effect could been masked (Zhou et al., 2021). |
| *LHFPL5* (*TMHS*) | LHFPL5 |  |  |  |  |  |
| **Description of protein** | |  |  |  |  |  |
| Probably acts as a mediator of force between the tip link and the MET complex | |  |  |  |  |  |
| **Associated disorders and models** | |  |  |  |  |  |
| DFNB67 *Hurry-scurry* (*hscy*) mouse | |  |  |  |  |  |
| **Gene** | **Encoded protein** | An alternative 3' splice site usage event at exon 4 was detected (Zhou et al., 2021). | The event introduces a frameshift and results in a shorter protein with a completely different C-terminal sequence (Zhou et al., 2021). | The frequency of the event was low, but was higher in OHCs when compared to IHCs (Zhou et al., 2021). |  |  |
| *TMIE* | TMIE |  |  |  |  |  |
| **Description of protein** | |  |  |  |  |  |
| Component of the MET complex closely associated with the channel | |  |  |  |  |  |
| **Associated disorders and models** | |  |  |  |  |  |
| DFNB6 *Spinner* (*sr*) mouse | |  |  |  |  |  |
| **Ion channels** | | | | | | |
| **Gene** | **Encoded protein** | **Splice variants** | **Structural consequences** | **Differential expression** | **Molecular properties** | **Relevance *in vivo*** |
| *CACNA1D* | Voltage-gated L-type Ca2^+^ channel (Cav1.3) subunit α1D | Mutually exclusive exons 8A and 8B have been described. | The exons both encode for the cytoplasmic end of the transmembrane helical segment S6 of domain I (Williams et al., 1992). The encoded peptide sequence of exon 8B differs from that of exon 8B by six amino acids. | Transcripts utilizing the two exons were both detected in mouse cochleae, but exon 8B was far more abundant than 8A in IHCs (Baig et al., 2011). |  | A mutation that affects only exon 8B underlies SANDD, a syndromic condition that includes deafness (Baig et al., 2011). Later, another mis-sense variant associated with the condition was identified, again in exon 8B (Liaqat et al., 2019). |
| **Description of protein** | |  |  |  |  |  |
| Ca2^+^ influx through the channel triggers glutamate release upon depolarization | |  |  |  |  |  |
| **Associated disorders and models** | |  |  |  |  |  |
| Sinoatrial node dysfunction and deafness (SANDD) | | Two additional exons, 11 and 32, have been detected in mouse brain samples, chromaffin cells, and cochlear preparations (Hofer et al., 2021). |  |  | When expressed *in vitro*, both isoforms were activated and inactivated at more negative voltages than did the canonical isoform (Hofer et al., 2021). |  |
|  |  | An alternative splice site usage event at exon 41 was detected (Shen et al., 2006). | C-terminal truncation removes the IQ domain necessary for CDI (Shen et al., 2006). | Truncated protein isoforms were more abundant in OHCs than in IHCs (Shen et al., 2006). | In *in vitro* experiments, CDI was eliminated in truncated channels (Shen et al., 2006). |  |
|  |  | An alternative exon, 42A, was found to be used in mutual exclusion with exon 42. Also, usage of an alternative splice site results in the production of exon 43S instead of the usual exon 43. Both events have been detected in the mouse organ of Corti and IHCs (Singh et al., 2008; Scharinger et al., 2015; Vincent et al., 2017). | Both AS events result in C-terminal truncation. In both short isoforms, the regulatory region downstream of the IQ domain is disrupted. This region is known to attenuate CDI through competitively inhibiting calmodulin from binding to the IQ domain. |  | Both short isoforms were activated at more negative voltages, and showed faster kinetics as well as stronger CDI (Singh et al., 2008; Bock et al., 2011; Tan et al., 2011; Huang et al., 2013). However, in one study that introduced a genetic modification predicted to disrupt the C-terminal domain in a similar manner, CDI in IHCs was reduced (Scharinger et al., 2015). | One study selectively blocked calcium currents through long isoforms in mice. It was concluded that fast-inactivating currents through short isoforms were responsible for fast fusion of the readily releasable pool, whereas slow-inactivating currents through long isoforms might contribute to steady vesicle replenishment (Vincent et al., 2017). |
|  |  | A long isoform with exon 44 spliced out was reported to be found in murine IHCs (Vincent et al., 2017). | The C-terminal regulatory domain is intact, although its distance towards the IQ domain is reduced. |  | The inactivation rate of the isoform was intermediate between those of the long isoform and the 42A or 43S isoforms (Tan et al., 2011). |  |
| **Gene** | **Encoded protein** | **Splice variants** | **Structural consequences** | **Differential expression** | **Molecular properties** | **Relevance *in vivo*** |
| *KCNMA1* | Pore-forming α subunit of the large-conductance Ca^2+^-activated K^+^ channel (BK channel) | Combinatorial inclusion of several alternative exons at 7 splice sites generates numerous splice variants. |  | The relative abundance of isoforms varies along the longitudinal axis of the auditory organ. This has been hypothesized to underlie electrical tuning. Consult the text for details. | Different channel isoforms possess different electrophysiological properties. |  |
| **Description of protein** | |  |  |  |  |  |
| Hyperpolarizes the HC to promote membrane potential oscillation | |  |  |  |  |  |
| **Associated disorders and models** | |  |  |  |  |  |
| Mice deficient for *Kcnma1* have normal hearing (Pyott et al., 2007) | |  |  |  |  |  |
| **Gene** | **Encoded protein** | Four splice variants, v1-4, arise from the mutually exclusive utilization of three alternative exons. Exons 10, 11, and 9 are utilized in variants v1, 2, and 3, respectively, and they are skipped altogether in v4. | The C-terminal region is affected, which is known to harbor domains responsible for calmodulin binding, endoplasmic reticulum retention, subcellular targeting in neurons, and protein kinase A phosphorylation (Xu et al., 2007). | The variants differ in tissue distribution and in expression along the tonotopic axis. This was considered as implying that the variants are differentially implicated in the pathogenesis of DFNB2. Consult the text for details. | Splice variants differ in voltage dependence and in modulation by calmodulin (Xu et al., 2007). |  |
| *KCNQ4* | Delayed rectifier K^+^ channel Kv7.4 |  |  |  |  |  |
| **Description of protein** | |  |  |  |  |  |
| Responsible mostly for the outward K^+^ current in OHCs, and partly in IHCs | |  |  |  |  |  |
| **Associated disorders and models** | |  |  |  |  |  |
| DFNA2 | |  |  |  |  |  |
| **Gene** | **Encoded protein** | Isoforms w-z are defined according to the combination of alternative exons inserted in splice site A. Isoforms a-c are defined likewise with regards to splice site C. | Splice site A encodes the first cytosolic loop domain, while splice site C encodes the C-terminal domain. | In the neonatal rat cochlea, w/a is the major isoform in HCs, z/b and z/c are predominant in spiral ganglion neurons, and w/a and w/c are the major splice forms in the stria vascularis (Chen et al., 2011). | The size of the splice site A insert seems to affect subcellular localization of the protein (Grati et al., 2006), so that isoforms w and z are targeted to the apical and basolateral membranes, respectively (Chicka and Strehler, 2003; Hill et al., 2006). In addition, the w/a isoform is less reactive to Ca^2+^ than w/b, z/a, or z/b isoforms (Ficarella et al., 2007). | A mutation responsible for DFNA82 was found to be located in one of the alternative exons included in the w isoform but not in the x, z isoforms (Smits et al., 2019). |
| *ATP2B2* | Plasma-membrane Ca^2+^-ATPase 2 (PMCA2) |  |  |  |  |  |
| **Description of protein** | |  |  |  |  |  |
| Removes majority of Ca^2+^ that enters stereocilia upon bundle deflection | |  |  |  |  |  |
| **Associated disorders and models** | |  |  |  |  |  |
| DFNA82 *Deafwaddler* (*dfw*), *Tommy*, and *Oblivion* (*Obl*) mice | |  |  |  |  |  |

| **Gene** | **Encoded protein** | **Splice variants** | **Structural consequences** | **Differential expression** | **Molecular properties** | **Relevance *in vivo*** |
| --- | --- | --- | --- | --- | --- | --- |
| *KCNN2* | Small-conductance Ca^2+^-activated K+ channel (SK2 channel) | A splice variant with a three-amino acid insert has been detected in the avian basilar papilla (Matthews et al., 2005) and in mice (Zhang et al., 2012). | The insert alters the conformation of the calmodulin-binding domain (Zhang et al., 2012). | In the chicken embryo, the proportion of transcripts utilizing the insert increases with time (Scholl et al., 2014) . | The resulting channel isoform is less sensitive to Ca^2+^ (Zhang et al., 2012). Also, its interaction with other proteins differs from that of the canonical isoform, and the interaction is differentially modulated by Ca^2+^ (Scholl et al., 2014). |  |
| **Description of protein** | |  |  |  |  |  |
| Ca^2+^ entry through α9/α10 nicotinic acetylcholine receptors at efferent synapses activates hyperpolarizing outward K+ currents through the SK2 channels | |  |  |  |  |  |
| **Miscellaneous** | | | | | | |
| **Gene** | **Encoded protein** | **Splice variants** | **Structural consequences** | **Differential expression** | **Molecular properties** | **Relevance *in vivo*** |
| *PCLO* | Piccolo | Alternative splicing has been detected in exons 16, 19, 21 and 22 (Fenster and Garner, 2002). Another splice variant, *Piccolino*, is generated by retention of intron 5 (Regus-Leidig et al., 2013). | The variants are predicted to encode shorter proteins that lack various functional domains located towards the C-terminal end (Fenster and Garner, 2002; Regus-Leidig et al., 2013). | *Piccolino* is expressed specifically in the ribbon synapses of the inner ear and the retina (Regus-Leidig et al., 2013). | Due to C-terminal truncation, Piccolino lacks its usual ability to interact with proteins such as Bassoon and Munc13 (Regus-Leidig et al., 2013). |  |
| **Description of protein** | |  |  |  |  |  |
| Component of the ribbon synapse | |  |  |  |  |  |
| **Associated disorders and models** | |  |  |  |  |  |
| *Pclo* knockout mice have normal hearing (Li et al., 2021) | |  |  |  |  |  |
| **Gene** | **Encoded protein** | In both mice and humans, exon skipping events have been documented in exons 6 and 47 and alternative 3'-splice site usage events in exon 31 (Yasunaga et al., 2000). | Exons 6 and 31 correspond to the first and fourth inter-C2 domains, respectively. Exon 47 encodes the C-terminal transmembrane domain and then introduces a stop codon. Skipping of exon 47 results in the translation of a highly similar amino acid sequence from exon 48 instead (Yasunaga et al., 2000). | Unlike in the brain, inclusion of exon 6 and skipping of exon 47 were predominant in the murine cochlea (Yasunaga et al., 2000). Exon 47 was selectively skipped in human cochleae as well (Choi et al., 2009). |  | Mutations located in exon 48 have been reported to be associated with nonsyndromic recessive auditory neuropathy (Yasunaga et al., 2000; Rodríguez-Ballesteros et al., 2003; Varga et al., 2003; Choi et al., 2009). |
| *OTOF* | Otoferlin |  |  |  |  |  |
| **Description of protein** | |  |  |  |  |  |
| Multi-C2 domain protein that acts as a Ca^2+^ sensor at ribbon synapses. Probably plays other roles as well in the exocytosis of synaptic vesicles | |  |  |  |  |  |
| **Associated disorders and models** | |  |  |  |  |  |
| DFNB9 AUNB1 *Pachanga* (*pga*) mouse | |  |  |  |  |  |

| **Gene** | **Encoded protein** | **Splice variants** | **Structural consequences** | **Differential expression** | **Molecular properties** | **Relevance *in vivo*** |
| --- | --- | --- | --- | --- | --- | --- |
| *TRIC* (*MARVELD2*) | Tricellulin | Splice variants a, a1, b and c have been documented in humans, whereas in mice variants a-e have been identified. (Transcripts with the same label do not correspond to each other.) (Riazuddin et al., 2006; Nayak et al., 2013). | In humans, *TRIC*-b encodes a protein that lacks a conserved cytosolic ZO-1-binding domain present in all other variants (Riazuddin et al., 2006). | In humans, *TRIC*-a was detected in fetal cochlear mRNA, whereas presence of *TRIC*-b was reported in the lung, mammary gland, and eye. *TRIC*-c was found in the lung (Riazuddin et al., 2006). |  |  |
| **Description of protein** | |  |  |  |  |  |
| Component of tricellular tight junctions broadly expressed in epithelia | |  |  |  |  |  |
| **Associated disorders and models** | |  |  |  |  |  |
| DFNB49 | |  |  |  |  |  |
| **Gene** | **Encoded protein** | Multiple splice variants have been detected in various tissues. In particular, one variant with a cryptic intron spliced out, *P2X2*-2, has been found in the inner ears of both rats and guinea pigs (Brändle et al., 1997; Housley et al., 1999). |  | In guinea pigs, *P2X2*-1 was the predominant transcript in the cochlear epithelium, while spiral ganglion neurons mainly expressed *P2X2*-2 (Housley et al., 1999). | When expressed in *Xenopus* oocytes, P2X2-2 showed faster desensitization rates than P2X2-1 (Brändle et al., 1997). |  |
| *P2RX2* | P2X2 |  |  |  |  |  |
| **Description of protein** | |  |  |  |  |  |
| Purinergic receptor expressed in IHCs | |  |  |  |  |  |
| **Associated disorders and models** | |  |  |  |  |  |
| DFNA41 | |  |  |  |  |  |
| **Gene** | **Encoded protein** | 3 mRNA splice variants have been identified in the rat cochlea (Sekine et al., 2010). In cultured human cells, 8 different splice variants have been identified (Kommareddi et al., 2007). Splicing patterns were not conserved between rats and human cells. | 2 long variants that differ in their 3'-UTR regions both encode the full length protein (Sekine et al., 2010). Multiple isoforms have been identified at the protein level (Ikezono et al., 2001; Ikezono et al., 2004), but it is unclear how or whether these correspond to splice variants. | Long variants were found across many types of tissues, whereas the short variant was expressed specifically in the inner ear (Sekine et al., 2010). |  |  |
| *COCH* | Cochlin |  |  |  |  |  |
| **Description of protein** | |  |  |  |  |  |
| An inner ear-specific protein of uncertain significance that constitutes 70% of the noncollagenous protein in the bovine inner ear | |  |  |  |  |  |
| **Associated disorders and models** | |  |  |  |  |  |
| DFNA9 | |  |  |  |  |  |

**Supplementary references**

Adato, A., Lefèvre, G., Delprat, B., Michel, V., Michalski, N., Chardenoux, S., et al. (2005). Usherin, the defective protein in Usher syndrome type IIA, is likely to be a component of interstereocilia ankle links in the inner ear sensory cells. *Hum. Mol. Genet.* 14(24)**,** 3921-3932. doi: 10.1093/hmg/ddi416.

Ahmed, Z.M., Goodyear, R., Riazuddin, S., Lagziel, A., Legan, P.K., Behra, M., et al. (2006). The Tip-Link Antigen, a Protein Associated with the Transduction Complex of Sensory Hair Cells, Is Protocadherin-15. *J. Neurosci.* 26(26)**,** 7022. doi: 10.1523/JNEUROSCI.1163-06.2006.

Ahmed, Z.M., Riazuddin, S., Aye, S., Ali, R.A., Venselaar, H., Anwar, S., et al. (2008). Gene structure and mutant alleles of PCDH15: nonsyndromic deafness DFNB23 and type 1 Usher syndrome. *Hum. Genet.* 124(3)**,** 215-223. doi: 10.1007/s00439-008-0543-3.

Aller, E., Jaijo, T., Beneyto, M., Nájera, C., Oltra, S., Ayuso, C., et al. (2006). Identification of 14 novel mutations in the long isoform of USH2A in Spanish patients with Usher syndrome type II. *J. Med. Genet.* 43(11)**,** e55. doi: 10.1136/jmg.2006.041764.

Baig, S.M., Koschak, A., Lieb, A., Gebhart, M., Dafinger, C., Nürnberg, G., et al. (2011). Loss of Cav1.3 (CACNA1D) function in a human channelopathy with bradycardia and congenital deafness. *Nat. Neurosci.* 14(1)**,** 77-84. doi: 10.1038/nn.2694.

Ballesteros, A., Yadav, M., Cui, R., Kurima, K., and Kachar, B. (2022). Selective binding and transport of protocadherin 15 isoforms by stereocilia unconventional myosins in a heterologous expression system. *Sci. Rep.* 12(1)**,** 13764. doi: 10.1038/s41598-022-17757-0.

Bhattacharya, G., Miller, C., Kimberling, W.J., Jablonski, M.M., and Cosgrove, D. (2002). Localization and expression of usherin: a novel basement membrane protein defective in people with Usher’s syndrome type IIa. *Hear. Res.* 163(1)**,** 1-11. doi: <https://doi.org/10.1016/S0378-5955(01)00344-6>.

Bock, G., Gebhart, M., Scharinger, A., Jangsangthong, W., Busquet, P., Poggiani, C., et al. (2011). Functional Properties of a Newly Identified C-terminal Splice Variant of Cav1.3 L-type Ca2+ Channels. *J. Biol. Chem.* 286(49)**,** 42736-42748. doi: <https://doi.org/10.1074/jbc.M111.269951>.

Brändle, U., Spielmanns, P., Osteroth, R., Sim, J., Surprenant, A., Buell, G., et al. (1997). Desensitization of the P2X2 receptor controlled by alternative splicing. *FEBS Lett.* 404(2-3)**,** 294-298. doi: <https://doi.org/10.1016/S0014-5793(97)00128-2>.

Chen, Q., Chu, H., Wu, X., Cui, Y., Chen, J., Li, J., et al. (2011). The expression of plasma membrane Ca(2+)-ATPase isoform 2 and its splice variants at sites A and C in the neonatal rat cochlea. *Int. J. Pediatr. Otorhinolaryngol.* 75(2)**,** 196-201. doi: 10.1016/j.ijporl.2010.10.033.

Chicka, M.C., and Strehler, E.E. (2003). Alternative Splicing of the First Intracellular Loop of Plasma Membrane Ca2+-ATPase Isoform 2 Alters Its Membrane Targeting. *J. Biol. Chem.* 278(20)**,** 18464-18470. doi: 10.1074/jbc.M301482200.

Choi, B., Ahmed, Z., Riazuddin, S., Bhinder, M., Shahzad, M., Husnain, T., et al. (2009). Identities and frequencies of mutations of the otoferlin gene (OTOF) causing DFNB9 deafness in Pakistan. *Clin. Genet.* 75(3)**,** 237-243. doi: <https://doi.org/10.1111/j.1399-0004.2008.01128.x>.

Dai, H., Zhang, X., Zhao, X., Deng, T., Dong, B., Wang, J., et al. (2008). Identification of five novel mutations in the long isoform of the USH2A gene in Chinese families with Usher syndrome type II. *Mol. Vis.* 14**,** 2067-2075.

Du, H., Zou, L., Ren, R., Li, N., Li, J., Wang, Y., et al. (2020). Lack of PDZD7 long isoform disrupts ankle-link complex and causes hearing loss in mice. *FASEB J* 34(1)**,** 1136-1149. doi: <https://doi.org/10.1096/fj.201901657RR>.

Fang, Q., Indzhykulian, A.A., Mustapha, M., Riordan, G.P., Dolan, D.F., Friedman, T.B., et al. (2015). The 133-kDa N-terminal domain enables myosin 15 to maintain mechanotransducing stereocilia and is essential for hearing. *eLife* 4**,** e08627. doi: 10.7554/eLife.08627.

Fenster, S.D., and Garner, C.C. (2002). Gene structure and genetic localization of the PCLO gene encoding the presynaptic active zone protein Piccolo. *Int. J. Dev. Neurosci.* 20(3-5)**,** 161-171. doi: <https://doi.org/10.1016/S0736-5748(02)00046-1>.

Ficarella, R., Di Leva, F., Bortolozzi, M., Ortolano, S., Donaudy, F., Petrillo, M., et al. (2007). A functional study of plasma-membrane calcium-pump isoform 2 mutants causing digenic deafness. *Proc. Natl. Acad. Sci.* 104(5)**,** 1516-1521. doi: 10.1073/pnas.0609775104.

Francis, S.P., Krey, J.F., Krystofiak, E.S., Cui, R., Nanda, S., Xu, W., et al. (2015). A Short Splice Form of Xin-Actin Binding Repeat Containing 2 (XIRP2) Lacking the Xin Repeats Is Required for Maintenance of Stereocilia Morphology and Hearing Function. *J Neurosci* 35(5)**,** 1999. doi: 10.1523/JNEUROSCI.3449-14.2015.

Grati, M.h., Aggarwal, N., Strehler, E.E., and Wenthold, R.J. (2006). Molecular determinants for differential membrane trafficking of PMCA1 and PMCA2 in mammalian hair cells. *J. Cell Sci.* 119(14)**,** 2995-3007. doi: 10.1242/jcs.03030.

Hill, J.K., Williams, D.E., LeMasurier, M., Dumont, R.A., Strehler, E.E., and Gillespie, P.G. (2006). Splice-Site A Choice Targets Plasma-Membrane Ca2+-ATPase Isoform 2 to Hair Bundles. *J Neurosci* 26(23)**,** 6172. doi: 10.1523/JNEUROSCI.0447-06.2006.

Hofer, N.T., Pinggera, A., Nikonishyna, Y.V., Tuluc, P., Fritz, E.M., Obermair, G.J., et al. (2021). Stabilization of negative activation voltages of Cav1.3 L-Type Ca2+-channels by alternative splicing. *Channels* 15(1)**,** 38-52. doi: 10.1080/19336950.2020.1859260.

Housley, G.D., Kanjhan, R., Raybould, N.P., Greenwood, D., Salih, S.G., Järlebark, L., et al. (1999). Expression of the P2X2 Receptor Subunit of the ATP-Gated Ion Channel in the Cochlea: Implications for Sound Transduction and Auditory Neurotransmission. *J Neurosci* 19(19)**,** 8377. doi: 10.1523/JNEUROSCI.19-19-08377.1999.

Huang, H., Yu, D., and Soong, T.W. (2013). C-Terminal Alternative Splicing of CaV1.3 Channels Distinctively Modulates Their Dihydropyridine Sensitivity. *Mol. Pharmacol.* 84(4)**,** 643. doi: 10.1124/mol.113.087155.

Ikezono, T., Omori, A., Ichinose, S., Pawankar, R., Watanabe, A., and Yagi, T. (2001). Identification of the protein product of the Coch gene (hereditary deafness gene) as the major component of bovine inner ear protein. *Biochim Biophys Acta Mol Basis Dis BBA - Mol Basis Dis* 1535(3)**,** 258-265. doi: <https://doi.org/10.1016/S0925-4439(00)00101-0>.

Ikezono, T., Shindo, S., Li, L., Omori, A., Ichinose, S., Watanabe, A., et al. (2004). Identification of a novel Cochlin isoform in the perilymph: insights to Cochlin function and the pathogenesis of DFNA9. *Biochem. Biophys. Res. Commun.* 314(2)**,** 440-446. doi: <https://doi.org/10.1016/j.bbrc.2003.12.106>.

Katsuno, T., Belyantseva, I.A., Cartagena-Rivera, A.X., Ohta, K., Crump, S.M., Petralia, R.S., et al. (2019). TRIOBP-5 sculpts stereocilia rootlets and stiffens supporting cells enabling hearing. *JCI Insight* 4(12). doi: 10.1172/jci.insight.128561.

Kawashima, Y., Géléoc, G.S.G., Kurima, K., Labay, V., Lelli, A., Asai, Y., et al. (2011). Mechanotransduction in mouse inner ear hair cells requires transmembrane channel–like genes. *J Clin Invest* 121(12)**,** 4796-4809. doi: 10.1172/JCI60405.

Khateb, S., Zelinger, L., Ben-Yosef, T., Merin, S., Crystal-Shalit, O., Gross, M., et al. (2012). Exome Sequencing Identifies a Founder Frameshift Mutation in an Alternative Exon of USH1C as the Cause of Autosomal Recessive Retinitis Pigmentosa with Late-Onset Hearing Loss. *PLoS One* 7(12)**,** e51566. doi: 10.1371/journal.pone.0051566.

Kitajiri, S.-i., Sakamoto, T., Belyantseva, I.A., Goodyear, R.J., Stepanyan, R., Fujiwara, I., et al. (2010). Actin-Bundling Protein TRIOBP Forms Resilient Rootlets of Hair Cell Stereocilia Essential for Hearing. *Cell* 141(5)**,** 786-798. doi: <https://doi.org/10.1016/j.cell.2010.03.049>.

Kommareddi, P.K., Nair, T.S., Raphael, Y., Telian, S.A., Kim, A.H., Arts, H.A., et al. (2007). Cochlin Isoforms and Their Interaction with CTL2 (SLC44A2) in the Inner Ear. *J Assoc Res Otolaryngol* 8(4)**,** 435-446. doi: 10.1007/s10162-007-0099-2.

Li, P., Lin, Z., An, Y., Lin, J., Zhang, A., Wang, S., et al. (2021). Piccolo is essential for the maintenance of mouse retina but not cochlear hair cell function. *Aging (Albany N. Y.)* 13(8)**,** 11678-11695. doi: 10.18632/aging.202861.

Li, Y., Lalwani, A.K., and Mhatre, A.N. (2007). Alternative Splice Variants of MYH9. *DNA Cell Biol.* 27(3)**,** 117-125. doi: 10.1089/dna.2007.0661.

Liaqat, K., Schrauwen, I., Raza, S.I., Lee, K., Hussain, S., Chakchouk, I., et al. (2019). Identification of CACNA1D variants associated with sinoatrial node dysfunction and deafness in additional Pakistani families reveals a clinical significance. *J. Hum. Genet.* 64(2)**,** 153-160. doi: 10.1038/s10038-018-0542-8.

Longo-Guess, C.M., Gagnon, L.H., Fritzsch, B., and Johnson, K.R. (2007). Targeted knockout and lacZ reporter expression of the mouse Tmhs deafness gene and characterization of the hscy-2J mutation. *Mamm. Genome* 18(9)**,** 646-656. doi: 10.1007/s00335-007-9049-x.

Matthews, T.M., Duncan, R.K., Zidanic, M., Michael, T.H., and Fuchs, P.A. (2005). Cloning and characterization of SK2 channel from chicken short hair cells. *Journal of Comparative Physiology A* 191(6)**,** 491-503. doi: 10.1007/s00359-005-0601-4.

McGee, T.L., Seyedahmadi, B.J., Sweeney, M.O., Dryja, T.P., and Berson, E.L. (2010). Novel mutations in the long isoform of the USH2A gene in patients with Usher syndrome type II or non-syndromic retinitis pigmentosa. *J. Med. Genet.* 47(7)**,** 499. doi: 10.1136/jmg.2009.075143.

Michalski, N., Michel, V., Caberlotto, E., Lefèvre, G.M., van Aken, A.F.J., Tinevez, J.-Y., et al. (2009). Harmonin-b, an actin-binding scaffold protein, is involved in the adaptation of mechanoelectrical transduction by sensory hair cells. *Pflugers Arch* 459(1)**,** 115. doi: 10.1007/s00424-009-0711-x.

Michel, V., Pepermans, E., Boutet de Monvel, J., England, P., Nouaille, S., Aghaie, A., et al. (2020). Interaction of protocadherin-15 with the scaffold protein whirlin supports its anchoring of hair-bundle lateral links in cochlear hair cells. *Sci. Rep.* 10(1)**,** 16430. doi: 10.1038/s41598-020-73158-1.

Narui, Y., and Sotomayor, M. (2018). Tuning Inner-Ear Tip-Link Affinity Through Alternatively Spliced Variants of Protocadherin-15. *Biochemistry* 57(11)**,** 1702-1710. doi: 10.1021/acs.biochem.7b01075.

Nayak, G., Lee, S.I., Yousaf, R., Edelmann, S.E., Trincot, C., Van Itallie, C.M., et al. (2013). Tricellulin deficiency affects tight junction architecture and cochlear hair cells. *J Clin Invest* 123(9)**,** 4036-4049. doi: 10.1172/JCI69031.

Ouyang, X., Xia, X., Verpy, E., Du, L., Pandya, A., Petit, C., et al. (2002). Mutations in the alternatively spliced exons of USH1C cause non-syndromic recessive deafness. *Hum. Genet.* 111(1)**,** 26-30. doi: 10.1007/s00439-002-0736-0.

Pepermans, E., Michel, V., Goodyear, R., Bonnet, C., Abdi, S., Dupont, T., et al. (2014). The CD2 isoform of protocadherin-15 is an essential component of the tip-link complex in mature auditory hair cells. *EMBO Mol. Med.* 6(7)**,** 984-992. doi: <https://doi.org/10.15252/emmm.201403976>.

Pyott, S.J., Meredith, A.L., Fodor, A.A., Vázquez, A.E., Yamoah, E.N., and Aldrich, R.W. (2007). Cochlear Function in Mice Lacking the BK Channel α, β1, or β4 Subunits. *J. Biol. Chem.* 282(5)**,** 3312-3324. doi: <https://doi.org/10.1074/jbc.M608726200>.

Regus-Leidig, H., Ott, C., Löhner, M., Atorf, J., Fuchs, M., Sedmak, T., et al. (2013). Identification and Immunocytochemical Characterization of Piccolino, a Novel Piccolo Splice Variant Selectively Expressed at Sensory Ribbon Synapses of the Eye and Ear. *PLoS One* 8(8)**,** e70373. doi: 10.1371/journal.pone.0070373.

Riazuddin, S., Ahmed, Z.M., Fanning, A.S., Lagziel, A., Kitajiri, S.-i., Ramzan, K., et al. (2006). Tricellulin Is a Tight-Junction Protein Necessary for Hearing. *Am J Hum Genet* 79(6)**,** 1040-1051. doi: <https://doi.org/10.1086/510022>.

Rodríguez-Ballesteros, M., del Castillo, F.J., Martín, Y., Moreno-Pelayo, M.A., Morera, C., Prieto, F., et al. (2003). Auditory neuropathy in patients carrying mutations in the otoferlin gene (OTOF). *Hum. Mutat.* 22(6)**,** 451-456. doi: 10.1002/humu.10274.

Scharinger, A., Eckrich, S., Vandael, D.H., Schönig, K., Koschak, A., Hecker, D., et al. (2015). Cell-type-specific tuning of Cav1.3 Ca(2+)-channels by a C-terminal automodulatory domain. *Front. Cell. Neurosci.* 9**,** 309. doi: 10.3389/fncel.2015.00309.

Scheffer, Déborah I., Zhang, D.-S., Shen, J., Indzhykulian, A., Karavitaki, K.D., Xu, Yichao J., et al. (2015). XIRP2, an Actin-Binding Protein Essential for Inner Ear Hair-Cell Stereocilia. *Cell Rep.* 10(11)**,** 1811-1818. doi: 10.1016/j.celrep.2015.02.042.

Scholl, E.S., Pirone, A., Cox, D.H., Duncan, R.K., and Jacob, M.H. (2014). Alternative splice isoforms of small conductance calcium-activated SK2 channels differ in molecular interactions and surface levels. *Channels (Austin)* 8(1)**,** 62-75. doi: 10.4161/chan.27470.

Sekine, K., Ikezono, T., Matsumura, T., Shindo, S., Watanabe, A., Li, L., et al. (2010). Expression of Cochlin mRNA Splice Variants in the Inner Ear. *Audiol Neurotol* 15(2)**,** 88-96. doi: 10.1159/000231634.

Shen, Y., Yu, D., Hiel, H., Liao, P., Yue, D.T., Fuchs, P.A., et al. (2006). Alternative Splicing of the CaV1.3 Channel IQ Domain, a Molecular Switch for Ca2+-Dependent Inactivation within Auditory Hair Cells. *J Neurosci* 26(42)**,** 10690-10699. doi: 10.1523/jneurosci.2093-06.2006.

Singh, A., Gebhart, M., Fritsch, R., Sinnegger-Brauns, M.J., Poggiani, C., Hoda, J.-C., et al. (2008). Modulation of Voltage- and Ca2+-dependent Gating of CaV1.3 L-type Calcium Channels by Alternative Splicing of a C-terminal Regulatory Domain. *J. Biol. Chem.* 283(30)**,** 20733-20744. doi: <https://doi.org/10.1074/jbc.M802254200>.

Smits, J.J., Oostrik, J., Beynon, A.J., Kant, S.G., de Koning Gans, P.A.M., Rotteveel, L.J.C., et al. (2019). De novo and inherited loss-of-function variants of ATP2B2 are associated with rapidly progressive hearing impairment. *Hum. Genet.* 138(1)**,** 61-72. doi: 10.1007/s00439-018-1965-1.

Tan, B.Z., Jiang, F., Tan, M.Y., Yu, D., Huang, H., Shen, Y., et al. (2011). Functional Characterization of Alternative Splicing in the C Terminus of L-type CaV1.3 Channels. *J. Biol. Chem.* 286(49)**,** 42725-42735. doi: <https://doi.org/10.1074/jbc.M111.265207>.

Västinsalo, H., Jalkanen, R., Dinculescu, A., Isosomppi, J., Geller, S., Flannery, J.G., et al. (2011). Alternative splice variants of the USH3A gene Clarin 1 (CLRN1). *Europ. J. Hum. Genet.* 19(1)**,** 30-35. doi: 10.1038/ejhg.2010.140.

van Wijk, E., Pennings, R.J.E., te Brinke, H., Claassen, A., Yntema, H.G., Hoefsloot, L.H., et al. (2004). Identification of 51 Novel Exons of the Usher Syndrome Type 2A (USH2A) Gene That Encode Multiple Conserved Functional Domains and That Are Mutated in Patients with Usher Syndrome Type II. *Am J Hum Genet* 74(4)**,** 738-744. doi: 10.1086/383096.

Varga, R., Kelley, P.M., Keats, B.J., Starr, A., Leal, S.M., Cohn, E., et al. (2003). Non-syndromic recessive auditory neuropathy is the result of mutations in the otoferlin (OTOF) gene. *J. Med. Genet.* 40(1)**,** 45-50. doi: 10.1136/jmg.40.1.45.

Vincent, P.F.Y., Bouleau, Y., Charpentier, G., Emptoz, A., Safieddine, S., Petit, C., et al. (2017). Different CaV1.3 Channel Isoforms Control Distinct Components of the Synaptic Vesicle Cycle in Auditory Inner Hair Cells. *J Neurosci* 37(11)**,** 2960. doi: 10.1523/JNEUROSCI.2374-16.2017.

Webb, S.W., Grillet, N., Andrade, L.R., Xiong, W., Swarthout, L., Della Santina, C.C., et al. (2011). Regulation of PCDH15 function in mechanosensory hair cells by alternative splicing of the cytoplasmic domain. *Development* 138(8)**,** 1607-1617. doi: 10.1242/dev.060061.

Williams, M.E., Feldman, D.H., McCue, A.F., Brenner, R., Velicelebi, G., Ellis, S.B., et al. (1992). Structure and functional expression of α1, α2, and β subunits of a novel human neuronal calcium channel subtype. *Neuron* 8(1)**,** 71-84. doi: <https://doi.org/10.1016/0896-6273(92)90109-Q>.

Wu, L., Pan, L., Zhang, C., and Zhang, M. (2012). Large protein assemblies formed by multivalent interactions between cadherin23 and harmonin suggest a stable anchorage structure at the tip link of stereocilia. *J. Biol. Chem.* 287(40)**,** 33460-33471. doi: 10.1074/jbc.M112.378505.

Xu, T., Nie, L., Zhang, Y., Mo, J., Feng, W., Wei, D., et al. (2007). Roles of Alternative Splicing in the Functional Properties of Inner Ear-specific KCNQ4 Channels. *J. Biol. Chem.* 282(33)**,** 23899-23909. doi: <https://doi.org/10.1074/jbc.M702108200>.

Xu, W., Dai, H., Lu, T., Zhang, X., Dong, B., and Li, Y. (2011). Seven novel mutations in the long isoform of the USH2A gene in Chinese families with nonsyndromic retinitis pigmentosa and Usher syndrome Type II. *Mol. Vis.* 17**,** 1537-1552.

Yamaguchi, S., Hamamura, M., and Otsuguro, K.-i. (2020). A Mechanosensitive Channel, Mouse Transmembrane Channel-Like Protein 1 (mTMC1) Is Translated from a Splice Variant mTmc1ex1 but Not from the Other Variant mTmc1ex2. *Int. J. Mol. Sci.* 21(18). doi: 10.3390/ijms21186465.

Yasunaga, S.i., Grati, M.h., Chardenoux, S., Smith, T.N., Friedman, T.B., Lalwani, A.K., et al. (2000). OTOF Encodes Multiple Long and Short Isoforms: Genetic Evidence That the Long Ones Underlie Recessive Deafness DFNB9. *Am J Hum Genet* 67(3)**,** 591-600. doi: <https://doi.org/10.1086/303049>.

Zhang, M., Abrams, C., Wang, L., Gizzi, A., He, L., Lin, R., et al. (2012). Structural Basis for Calmodulin as a Dynamic Calcium Sensor. *Structure* 20(5)**,** 911-923. doi: <https://doi.org/10.1016/j.str.2012.03.019>.

Zhao, B., Wu, Z., Grillet, N., Yan, L., Xiong, W., Harkins-Perry, S., et al. (2014). TMIE Is an Essential Component of the Mechanotransduction Machinery of Cochlear Hair Cells. *Neuron* 84(5)**,** 954-967. doi: <https://doi.org/10.1016/j.neuron.2014.10.041>.

Zhou, Z., Yu, X., Jiang, B., Feng, W., Tian, Y., Liu, Z., et al. (2021). Alternative Splicing of Three Genes Encoding Mechanotransduction-Complex Proteins in Auditory Hair Cells. *eneuro* 8(1)**,** ENEURO.0381-0320.2020. doi: 10.1523/ENEURO.0381-20.2020.

Zou, J., Zheng, T., Ren, C., Askew, C., Liu, X.-P., Pan, B., et al. (2014). Deletion of PDZD7 disrupts the Usher syndrome type 2 protein complex in cochlear hair cells and causes hearing loss in mice. *Hum. Mol. Genet.* 23(9)**,** 2374-2390. doi: 10.1093/hmg/ddt629.
